# circRNA-AKT1 Sequesters miR-942-5p to Upregulate AKT1 and Promote Cervical Cancer Progression

**DOI:** 10.1016/j.omtn.2020.01.003

**Published:** 2020-01-16

**Authors:** Rongying Ou, Laiming Mo, Huijing Tang, Shaolong Leng, Haiyan Zhu, Liang Zhao, Yi Ren, Yunsheng Xu

**Affiliations:** 1 Laboratory for Advanced Interdisciplinary Research, Institutes of Translational Medicine, The First Affiliated Hospital of Wenzhou Medical University, Wenzhou, China; 2 Department of Gynaecology and Obstetrics, The First Affiliated Hospital of Wenzhou Medical University, Wenzhou, China; 3 Clinical Laboratory, The Seventh Affiliated Hospital, Sun Yat-sen University, Shenzhen, China; 4 Department of Dermatovenereology, The Seventh Affiliated Hospital, Sun Yat-sen University, Shenzhen, China; 5 Department of Biomedical Sciences, Florida State University College of Medicine, Tallahassee, FL, USA

**Keywords:** circ-AKT1, miR-942-5p, AKT1, cervical cancer

## Abstract

Statistics show that the prognosis of cervical cancer (CC) is poor, and the death rate of CC in advanced stage has been rising in recent years. Increasing evidence has demonstrated that circular RNAs (circRNAs) serve as promising biomarkers in human cancers, including CC. The present study planned to find out the circRNA involved in CC and to explore its regulatory mechanism in CC. We discovered the new circRNA, circ-0033550, upregulated in CC. Its associated gene was AKT (also known as protein kinase B) serine/threonine kinase 1 (AKT1), so we renamed circ-0033550 as circ-AKT1. We confirmed the high expression of circ-AKT1 in CC samples and cell lines, as well as the circle structure of circ-AKT1. Functionally, gain- and loss-of-function experiments indicated that circ-AKT1 and AKT1 promoted CC cell proliferation and invasion. Moreover, circ-AKT1 and AKT1 were induced by transforming growth factor beta (TGF-β) and facilitated EMT (epithelial-mesenchymal transition) in CC. Mechanically, we illustrated that circ-AKT1 upregulated AKT1 by sponging miR-942-5p. Rescue assays confirmed the role of the circ-AKT1/miR-942-5p/AKT1 axis in CC progression. *In vivo* assays validated that circ-AKT1 promoted tumor growth in CC. Overall, circRNA-AKT1 sequestered miR-942-5p to upregulate AKT1 and promote CC progression, which may offer a new molecular target for the treatment improvement of CC.

## Introduction

Cervical cancer (CC) is a class of cancer in the gynecologic system, taking up a large proportion of global cancer-associated mortality.^1^^,^^2^ Despite that the past three decades have witnessed a decrease in the incidence and death rate of CC owing to the wide application of Pap smear screening tests, CC patients at advanced stage remain suffering from a dismal prognosis.^3^^,^^4^ Therefore, deep investigation on the mechanism behind CC progression is beneficial to the improvement of CC treatment and prognosis.

Circular RNAs (circRNAs) are a subset of non-coding RNAs (ncRNAs) incapable of translating proteins.^5^^,^^6^ Initially referred to as splicing errors,^7^ circRNAs have a structure of covalently closed loop short of 3′ and 5′ ends.^8^^,^^9^ However, more and more studies have demonstrated the implication of circRNAs with aberrant expression in the progression of cancers,^10^^,^^11^ including in CC.^12^ circRNAs have been reported to affect tumor-related cellular activities, such as cell proliferation, invasion, as well as epithelial-mesenchymal transition (EMT) process.^13^, ^14^, ^15^, ^16^ circRNA-0033550 is a newly found circRNA by our study. It aroused our interest not only because of its upregulated expression in CC samples discovered by microarray, but also because of the associated relation between circRNA-0033550 and AKT (protein kinase B) serine/threonine kinase 1 (AKT1) gene. Therefore, we renamed circRNA-0033550 as circ-AKT1 and focused on exploring its role in CC.

AKT1, a serine/threonine kinase with high conservative property, is a pivotal component involved in the phosphatidylinositol 3-kinase (PI3K)/AKT pathway.^17^^,^^18^ Moreover, AKT1 is responsible for several cellular processes, such as proliferation, migration, and invasion of cells.^19^, ^20^, ^21^ Additionally, the involvement of AKT1 in CC has been demonstrated by several studies.^22^^,^^23^ However, the correlation of AKT1 with circ-AKT1 has never been investigated.

Mechanistic research has demonstrated that circRNAs can work through sponging microRNAs (miRNAs) and regulating downstream gene expression so as to regulate human cancers,^24^^,^^25^ including CC.^12^ miRNAs, defined as small ncRNAs with 19–24 nt, are proved to be essential modulators of pathogenesis and development of numerous cancers,^26^ including CC.^27^^,^^28^ Via targeting the 3′ untranslated region (3′ UTR) of messenger RNAs (mRNAs) to cause translation repression or degradation, miRNAs can silence the expressions of target mRNAs.^29^^,^^30^ miR-942-5p has been reported to exert suppressive function in Kaposi’s sarcoma-associated herpesvirus (KSHV)-associated malignancies.^31^ However, its function has never been explored in CC, and its relation with circ-AKT1 and AKT1 remains elusive.

The purpose of our study is to find out the role and mechanism of circ-AKT in CC progression, and we conclude that circRNA-AKT1 sequesters miR-942-5p to upregulate AKT1 and promote CC progression.

## Results

### circ-AKT1 Was Upregulated in CC

**Figure 1:**
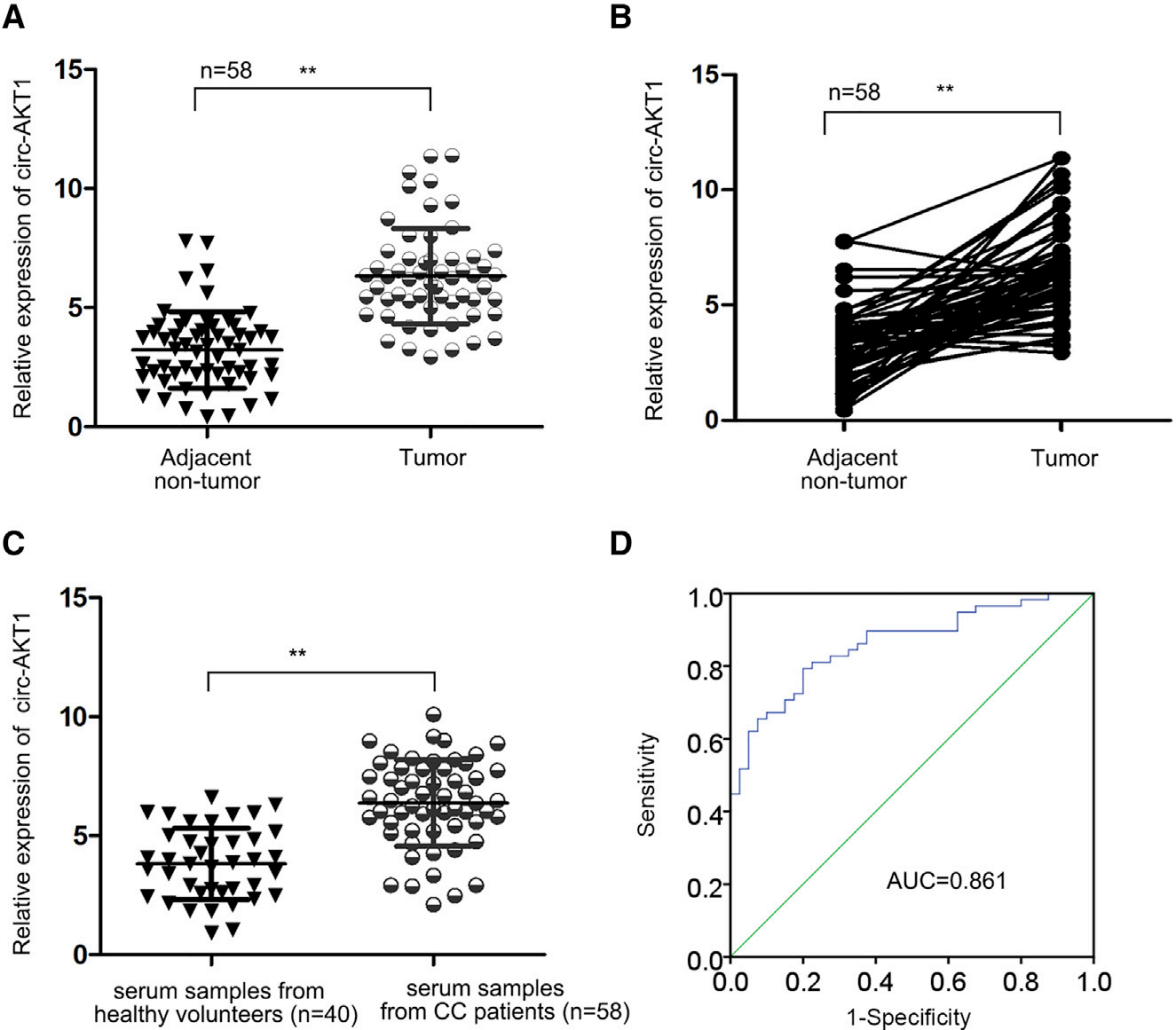
circ-AKT1 Was Upregulated in CC (A and B) Quantitative real-time RT-PCR detected the relative expression of circ-AKT1 in 58 CC tissues and 58 matched non-cancerous tissues. (C) Quantitative real-time RT-PCR measured the relative expression of circ-AKT1 in the serum of CC patients (n = 58) and in that of healthy volunteers (n = 40). (D) ROC curve displayed the diagnostic performance of circ-AKT1 for CC patients. **p < 0.01.

First, we identified that circRNAs participated in CC through circRNA microarray. As shown by the heatmap (Figures S1A and S1B), 1,842 circRNAs were upregulated and 1,620 circRNAs were downregulated in CC tissues (n = 3) compared with the matched adjacent non-cancerous tissues (n = 3), and the top 50 upregulated circRNAs were picked up for further research (Table S2). From the 50 upregulated circRNAs, we selected circ-AKT1, whose annotated gene was AKT1. Besides, AKT1 has been widely reported to be a frequently activated kinase in cancers,^23^^,^^32^^,^^33^ exerting important functions in regulating cellular activities such as cell proliferation and invasion.^19^, ^20^, ^21^ We then confirmed that circ-AKT1 was highly expressed in CC samples in contrast with the paired non-tumor samples (Figures 1A and 1B). Moreover, we also measured that circ-AKT1 was highly expressed in the serum of 58 CC patients, and low expressed in that of 40 healthy volunteers via quantitative real-time reverse transcription polymerase chain reaction (RT-PCR) (Figure 1C). Subsequently, ROC (receiver operating characteristic) curves were made, and the results indicated that circ-AKT1 may have diagnostic value for CC patients (Figure 1D). Therefore, these data confirmed that circ-AKT1 was upregulated in CC, indicating that circ-AKT1 might participate in CC.

### circ-AKT1 Was a Bona Fide circRNA

**Figure 2:**
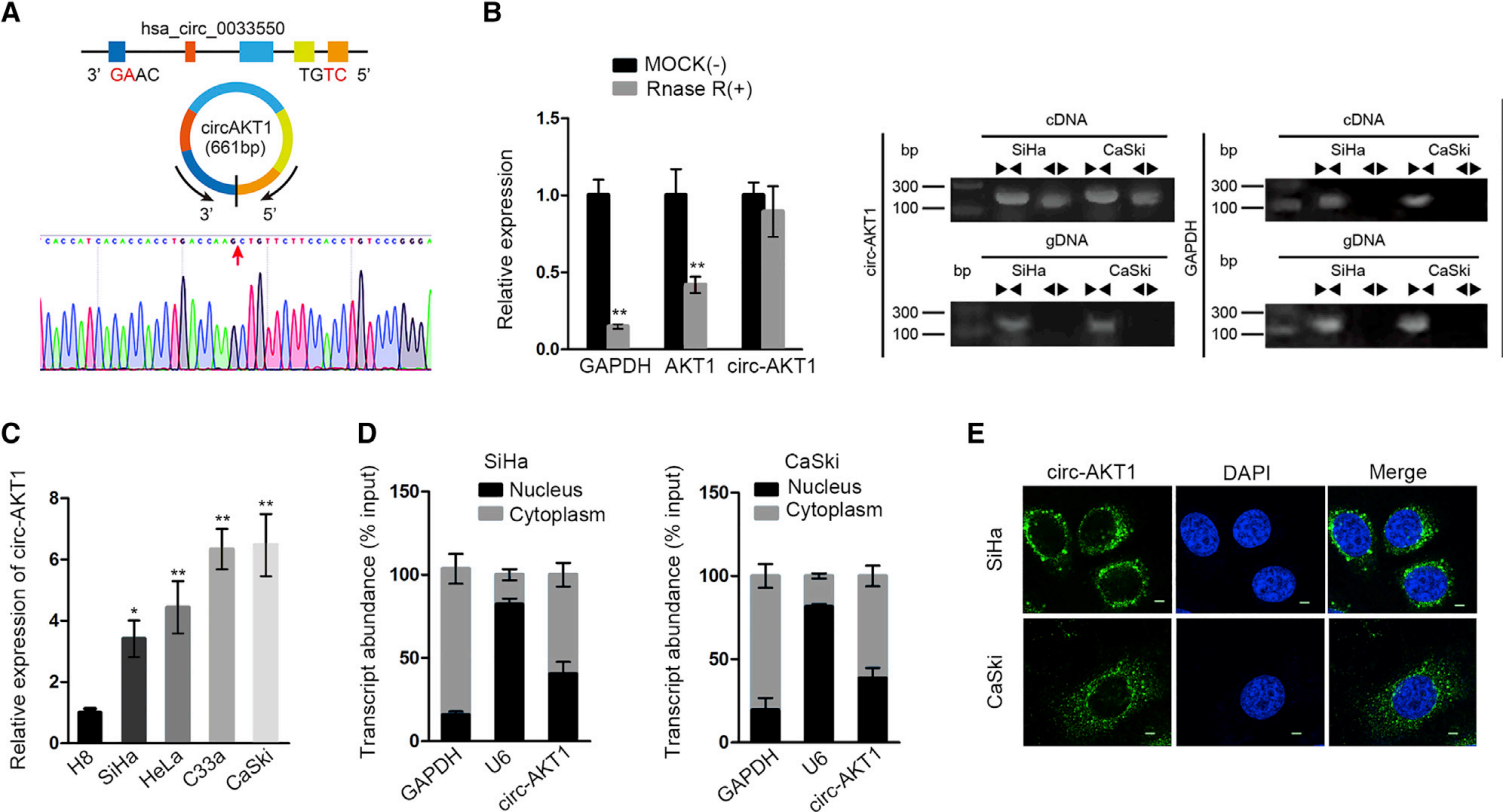
circ-AKT1 Was a Bona Fide circRNA (A) circRNA sequencing analysis displayed the transcription process of circ-AKT1. (B) Quantitative real-time RT-PCR measured relative expression of circ-AKT1 and AKT1 in the RNase R-treated group. GAPDH was the negative control (left); PCR results measured the amplification primer of circ-AKT1 in cDNA and gDNA (right). (C) Relative expression of circ-AKT1 in CC cell lines was tested by quantitative real-time RT-PCR. (D) Subcellular fractionation assay detected transcript abundance of circ-AKT1 in cytoplasm and nucleus of SiHa and CaSki cells. (E) FISH staining confirmed the expression of circ-AKT1 in cytoplasm (scale bars, 10 μm). *p < 0.05, **p < 0.01.

Next, we tried to examine circular characteristics of circ-AKT1. We confirmed through circRNA sequencing that circ-AKT1 was backspliced from AKT1 gene, ranging from the 9th exon to the 13th exon of the host, which contains five exons (Figure 2A). Also, we found that, compared with AKT1, circAKT1 was resistant to the digestion by RNase R (Figure 2B, left), and that circ-AKT1 amplified by divergent primer was identified in complementary DNA (cDNA) rather than genomic DNA (gDNA) (Figure 2B, right). Besides, in Figure S2A, northern blot assay detected that the RNA expressions of GAPDH and AKT1 were decreased in RNase R treatment, whereas that of circ-AKT1 was not affected. Additionally, overexpression efficiency of circ-AKT1 was detected with or without Alu treatment (Figure S2B). Then we confirmed the elevated expression of circ-AKT1 in four CC cell lines (Figure 2C). Additionally, we examined the cellular localization of circ-AKT1. Subcellular fractionation analysis and fluorescence *in situ* hybridization (FISH) staining validated the concentrating distribution of circ-AKT1 in CC cell cytoplasm (Figures 2D and 2E). In general, these findings confirmed the circular structure and post-transcriptional regulation possibility of circ-AKT1 in CC.

### circ-AKT1 Promoted Cell Proliferation and Invasion in CC

**Figure 3:**
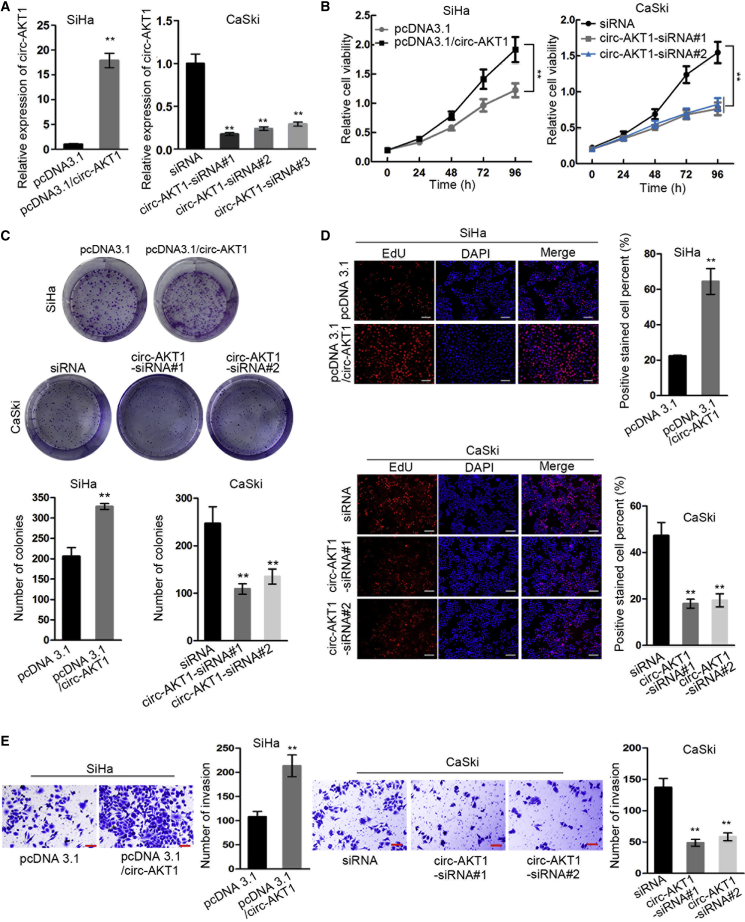
circ-AKT1 Promoted Cell Proliferation and Invasion in CC (A) Quantitative real-time RT-PCR detected relative expression of circ-AKT1 in pcDNA3.1/circ-AKT1-transfected SiHa cells and circ-AKT1-siRNA#1-, circ-AKT1-siRNA#2-, or circ-AKT1-siRNA#3-transfected CaSki cells. (B) CCK-8 detected SiHa and CaSki cell viability in differently transfected conditions. (C) Colony formation assay measured colony number of transfected SiHa and CaSki cells. (D) EdU assay detected positive stained cell percent when overexpressing or knocking down circ-AKT1 (scale bars, 100 μm). (E) Transwell invasion assay detected the invasive ability of SiHa and CaSki cells upon circ-AKT1 overexpression and knockdown (scale bars, 60 μm). **p < 0.01.

Then we explored the functional role of circ-AKT1 in CC through gain- and loss-of-function experiments. Because previously we found that circ-AKT1 presented the lowest expression in SiHa cells and highest in CaSki cells among four CC cells, we overexpressed circ-AKT1 in SiHa cells and knocked down circ-AKT1 in CaSki cells. Quantitative real-time RT-PCR results validated the upregulation of circ-AKT1 by pcDNA3.1/circ-AKT1 and the knockdown of circ-AKT1 by three specific siRNAs. In addition, siRNA#1 and siRNA#2 exhibited better knockdown efficiency (Figure 3A). Therefore, we used siRNA#1 and siRNA#2 for loss-of-function experiments. Results of Cell Counting Kit-8 (CCK-8) and colony formation assays displayed that CC cell proliferation was facilitated by circ-AKT1 overexpression but retarded by circ-AKT1 knockdown (Figures 3B and 3C). Also, 5-ethynyl-2′-deoxyuridine (EdU) assay demonstrated that the proliferative cells were increased by circ-AKT1 overexpression and were decreased by circ-AKT1 knockdown (Figure 3D). Transwell invasion assay showed that overexpression of circ-AKT1 enhanced invasive ability of CC cells, and that knockdown of circ-AKT1 led to opposite results (Figure 3E). On the whole, these data suggested that circ-AKT1 promoted cell proliferation and invasion in CC.

### AKT1 Was Upregulated in CC and Promoted Proliferation and Invasion

**Figure 4:**
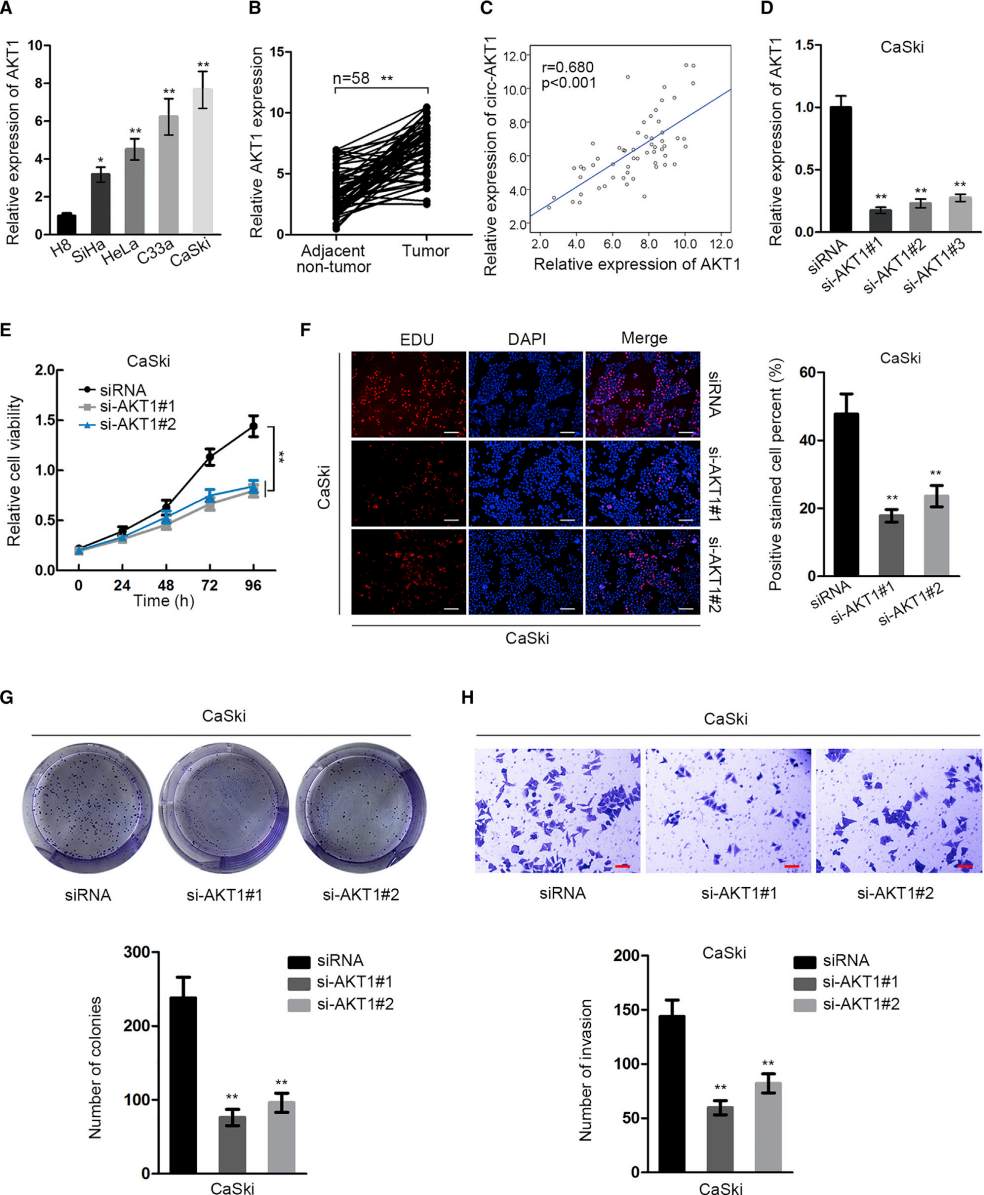
AKT1 Was Upregulated in CC Cells and Promoted Proliferation and Invasion (A and B) Quantitative real-time RT-PCR detected relative expression of AKT1 in CC cell lines and tissues. (C) Spearman’s correlation analysis was used to confirm the correlation between circ-AKT1 and AKT1 expressions in CC samples. (D) Quantitative real-time RT-PCR measured relative expression of AKT1 in si-AKT1#1-, si-AKT1#2-, and si-AKT1#3-transfected CaSki cells. (E) CCK-8 tested cell viability of CaSki cells. (F) EdU assay detected positive stained cell percent (scale bars, 100 μm). (G) Colony formation assay measured colony number in differently transfected groups. (H) Transwell invasion tested the number of invasion SiHa and CaSki cells in response to the transfection of si-AKT1#1 or si-AKT1#2 compared with control (scale bars, 60 μm). *p < 0.05, **p < 0.01.

Additionally, we tested the effect of AKT1 on CC development. We confirmed the high expression of AKT1 in CC cell lines and tissues (Figures 4A and 4B). In CC samples, we verified the positive correlation between AKT1 and circ-AKT1 (Figure 4C). We then knocked down AKT1 in CaSki cells, which was confirmed by quantitative real-time RT-PCR results (Figure 4D). We chose si-AKT1#1 and si-AKT1#2 for subsequent assays because they present better knockdown efficiency. CCK-8 and EdU assays illustrated that silencing AKT1 attenuated proliferative capacity of CC cells (Figures 4E and 4F). Also, knockdown of AKT1 decreased the colony number in CaSki cells (Figure 4G). Transwell invasion assay revealed that CC cells with AKT1 depletion presented weaker invasive ability than control (Figure 4H). In summary, the results above suggested that AKT1, positively correlated with circ-AKT1, was upregulated in CC cells and promoted cell proliferation and invasion.

### TGF-β-Induced circ-AKT1 Upregulated AKT1 and Promoted EMT in CC

**Figure 5:**
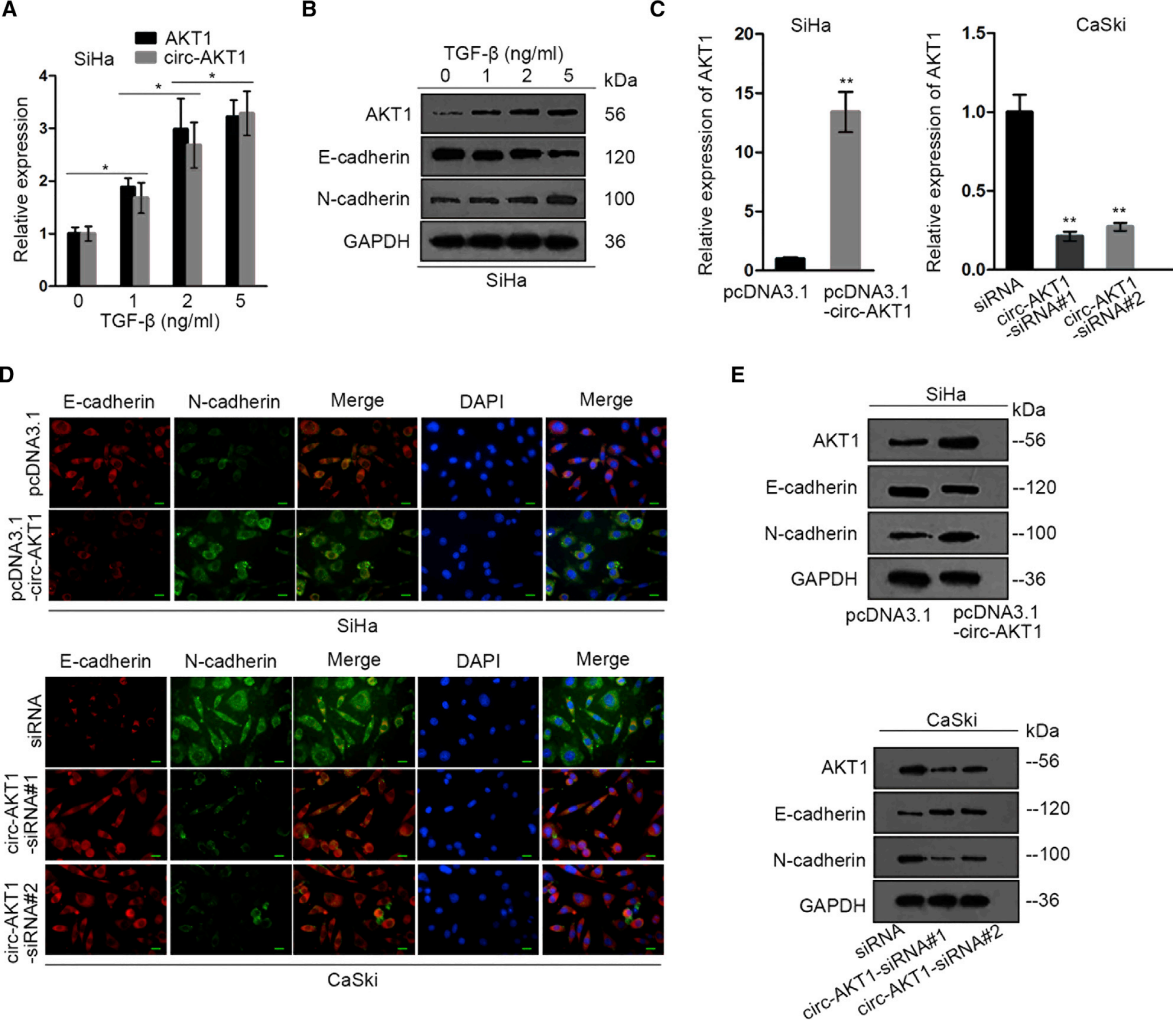
TGF-β-Induced circ-AKT1 Upregulated AKT1 and Promoted EMT in CC (A) Quantitative real-time RT-PCR detected the expressions of circ-AKT1 and AKT1 in SiHa cells treated with TGF-β at the dose of 0, 1, 2, and 5 ng/mL. (B) Western blot assay displayed the expressions of AKT1, E-cadherin, and N-cadherin in SiHa cells treated with TGF-β at the dose of 0, 1, 2, and 5 ng/mL. (C) Quantitative real-time RT-PCR measured the expression of AKT1 in SiHa and CaSki cells upon circ-AKT1 overexpression and knockdown. (D) IF (immunofluorescence) staining detected the fluorescence intensities of E-cadherin and N-cadherin in SiHa and CaSki cells upon circ-AKT1 overexpression and knockdown (scale bars, 40 μm). (E) Western blot assay tested the expressions of AKT1, E-cadherin, and N-cadherin in SiHa and CaSki cells upon circ-AKT1 overexpression and knockdown. *p < 0.05, **p < 0.01.

It has been known that transforming growth factor beta (TGF-β) is an upstream inducer of AKT1;^34^^,^^35^ in addition, quantitative real-time RT-PCR detected that the expression of TGF-β was more upregulated in CC tissues and cells compared with that in corresponding control groups (Figures S3A and 3B). Therefore, we probed whether TGF-β was also responsible for the upregulation of circ-AKT1 in CC. After treating SiHa cells with TGF-β at the dose of 0, 1, 2, and 5 ng/mL, we detected the expressions of circ-AKT1 and AKT1. The results showed that the expressions of both AKT1 and circ-AKT1 were dose dependently increased by TGF-β (Figure 5A). Previous reports have illustrated that AKT1 regulated EMT progress in human cancers^36^^,^^37^ and facilitated EMT progress, which enhanced migratory and invasive capacities of cancer cells.^38^ Therefore, we examined the effect of TGF-β on the expressions of EMT markers. Levels of AKT1 and mesenchymal marker N-cadherin were increased, whereas that of epithelial marker E-cadherin was decreased by TGF-β in a dose-dependent manner (Figure 5B). Furthermore, we detected the effect of circ-AKT1 on AKT1 expression. Results of quantitative real-time RT-PCR showed the increased AKT1 level upon circ-AKT1 overexpression and the decreased AKT1 level upon circ-AKT1 knockdown (Figure 5C). Later, immunofluorescence (IF) staining exhibited that overexpression of circ-AKT1 increased N-cadherin and decreased E-cadherin expression, whereas silencing circ-AKT1 exhibited the opposite effects (Figure 5D). The same results were observed through western blot analysis as well (Figure 5E). Generally speaking, these results indicated that TGF-β-induced circ-AKT1 upregulated AKT1 and promoted EMT in CC.

### circ-AKT1 Sequestered miR-942-5p to Regulate AKT1 in CC

**Figure 6:**
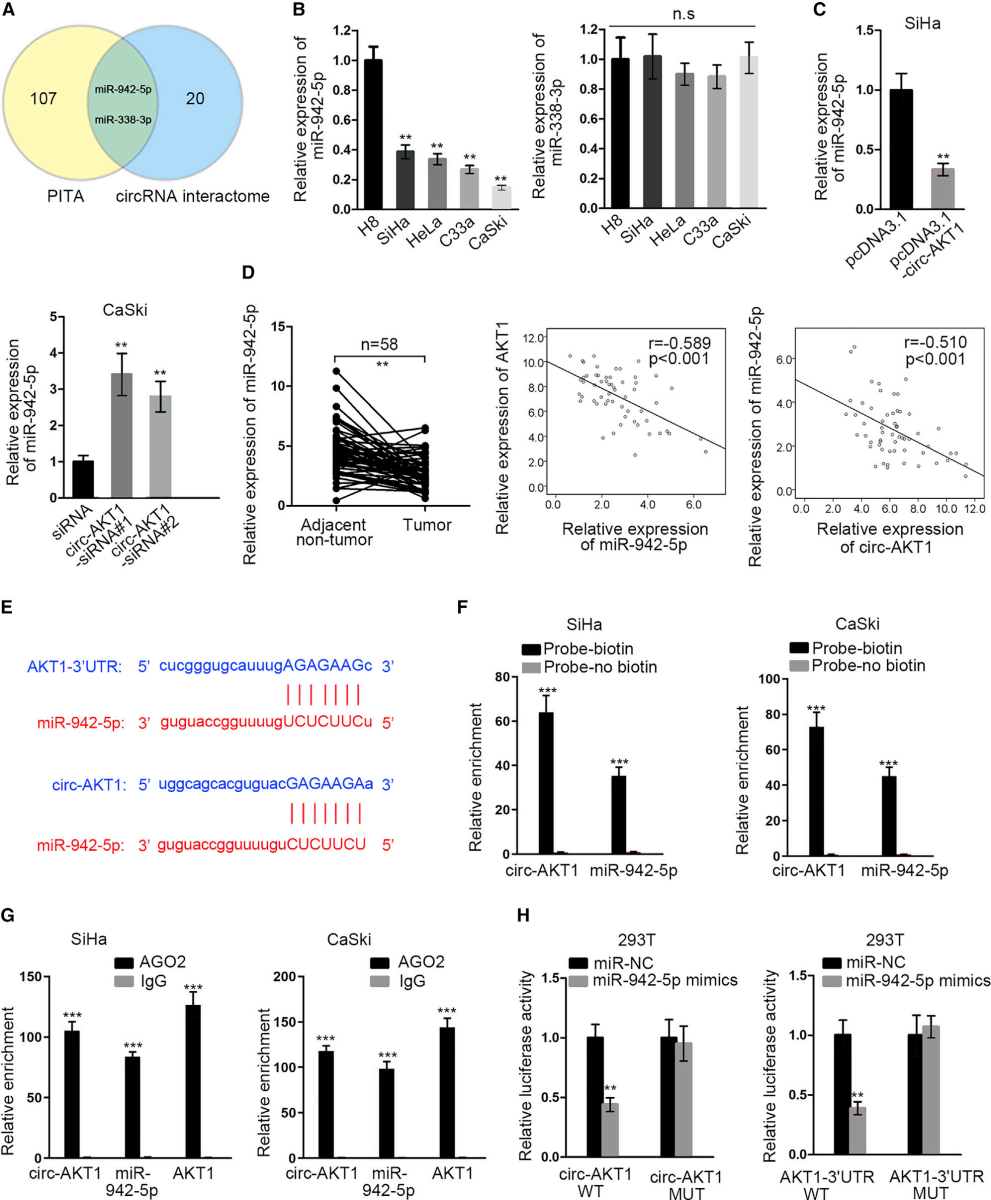
circ-AKT1 Sequestered miR-942-5p to Regulate AKT1 in CC (A) The Venn diagram showed that miR-942-5p and miR-338-3p were in the intersection of prediction results of PITA and CircInteractome databases. (B) Quantitative real-time RT-PCR detected relative expression of miR-942-5p and miR-338-3p in CC cell lines. (C) Quantitative real-time RT-PCR was used to detect the expression of miR-942-5p upon circ-AKT1 overexpression and knockdown in SiHa and CaSki cells. (D) Quantitative real-time RT-PCR measured the expression of miR-942-5p in CC samples (left). Spearman’s correlation curve showed the correlation of miR-942-5p with circ-AKT1 and AKT1 (right). (E) The binding sites between AKT1 and miR-942-5p, and the binding sites between circ-AKT1 and miR-942-5p were predicted by starBase. (F) Pull-down assay detected the input recovery of circ-AKT1 and miR-942-5p in SiHa and CaSki cells. (G) RIP assays measured the input recovery of circ-AKT1, miR-942-5p, and AKT1 in SiHa and CaSki cells. (H) Luciferase reporter assay was used to examine the luciferase activity of circ-AKT1-WT and circ-AKT1-MUT in transfected 293T cells. **p < 0.01, ***p < 0.001.

Plentiful studies have demonstrated that circRNAs are able to function through competing endogenous RNA (ceRNA) mechanism in cancers, whereby they could sequester miRNAs to induce downstream gene expression.^12^ Therefore, we planned to confirm whether circ-AKT1 could regulate AKT1 expression through targeting miRNAs. We searched for the miRNAs potentially interacting with both circ-AKT1 and AKT1. We found 107 putative miRNAs targeting AKT1 from PITA, and 20 miRNAs targeted by circ-AKT1 from CircInteractome. The intersection of the findings from the two databases contained two miRNAs (miR-942-5p and miR-338-3p) (Figure 6A). To detect the involvement of these two miRNAs in CC, we examined their expressions in cell lines. As a result, only miR-942-5p was downregulated in CC cell lines compared with the normal cell line (Figure 6B), indicating that miR-942-5p was associated with CC. Therefore, we focused on the investigation of miR-942-5p in the following experiments. Quantitative real-time RT-PCR validated that overexpression of circ-AKT1 reduced the level of miR-942-5p, whereas knockdown of circ-AKT1 resulted in the opposite conclusion (Figure 6C). Additionally, miR-942-5p was downregulated in CC samples and negatively correlated with circ-AKT1 and AKT1 expressions (Figure 6D).

Subsequently, we examined the interaction of miR-942-5p with circ-AKT1 and AKT1. The binding sequences on circ-AKT1 and AKT1 for miR-942-5p were presented in Figure 6E. RNA pull-down assay revealed that miR-942-5p could be pulled down by the biotin-circ-AKT1 probe instead of the no biotin probe (Figure 6F). RNA immunoprecipitation (RIP) assay validated that circ-AKT1, miR-942-5p, and AKT1 could be co-immunoprecipitated by anti-Ago2 (Figure 6G). Luciferase reporter assay demonstrated that overexpressing miR-942-5p decreased the luciferase activity of circ-AKT WT (wild-type) and AKT1 WT. Meanwhile, that of circ-AKT MUT (mutant) and AKT1 MUT was not affected (Figure 6H). Therefore, these results suggested that circ-AKT1 sequestered miR-942-5p to regulate AKT1 in CC.

### circ-AKT1 Regulated CC Progression through the miR-942-5p/AKT1 Axis

**Figure 7:**
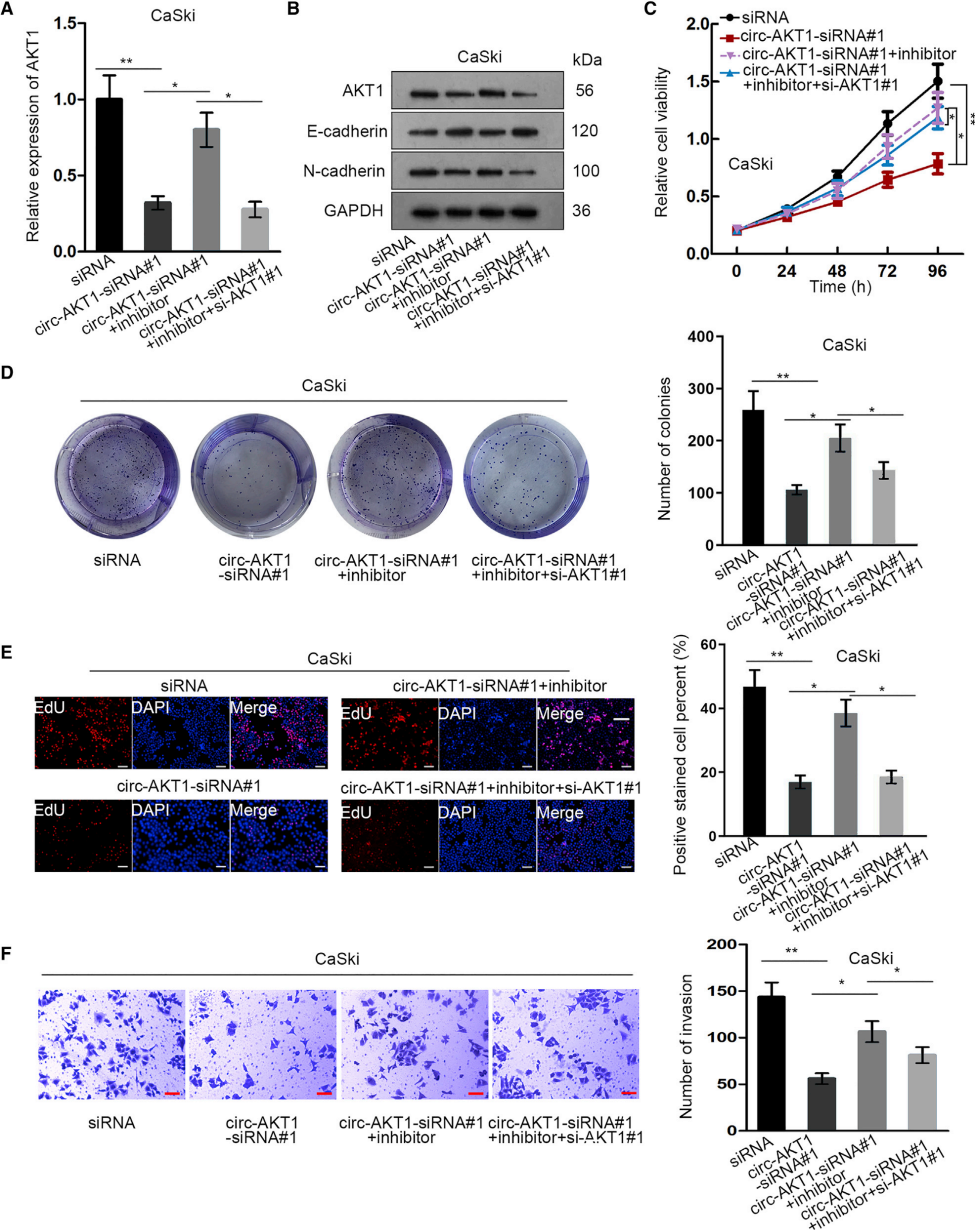
circ-AKT1 Regulated CC Progression through the miR-942-5p/AKT1 Axis CaSki cells were transfected with siRNA, circ-AKT1-siRNA#1, circ-AKT1-siRNA#1 + miR-942-5p inhibitor, or circ-AKT1-siRNA#1 + miR-942-5p inhibitor + si-AKT1#1 for subsequent assays. (A) Quantitative real-time RT-PCR detected AKT1 expression in each group. (B) Western blot assay measured the expressions of AKT1, E-cadherin, and N-cadherin in each group. (C) CCK-8 tested cell viability of CC cells in each group. (D) Colony formation assay measured colony number in differently transfected groups. (E) EdU assay detected positive stained cell percent in each group (scale bars, 100 μm). (F) Transwell invasion assay was used to detect the invasive ability of CC cells in each group (scale bars, 60 μm). *p < 0.05, **p < 0.01.

In order to explore whether circ-AKT1 regulated CC progression through the miR-942-5p/AKT1 axis, we performed rescue assays. AKT1 expression decreased by circ-AKT1 knockdown was restored by miR-942-5p inhibitor, but such restoration could be impaired by silencing AKT1 in CaSki cells (Figure 7A). Western blot analyses showed that the increased expression of E-cadherin or decreased expressions of N-cadherin and AKT1 caused by circ-AKT1 silence could be reversed by miR-942-5p inhibitor, but such reversing effect could be counteracted by AKT1 knockdown (Figure 7B). Moreover, the CC cell proliferation and invasion attenuated by circ-AKT1 knockdown were restored by miR-942-5p inhibition, and similarly, such restoration role could be abrogated by AKT1 depletion (Figures 7C–7F). Altogether, it was indicated that circ-AKT1 regulated CC progression through the miR-942-5p/AKT1 axis.

### circ-AKT1 Facilitated CC Progression *In Vivo*

**Figure 8:**
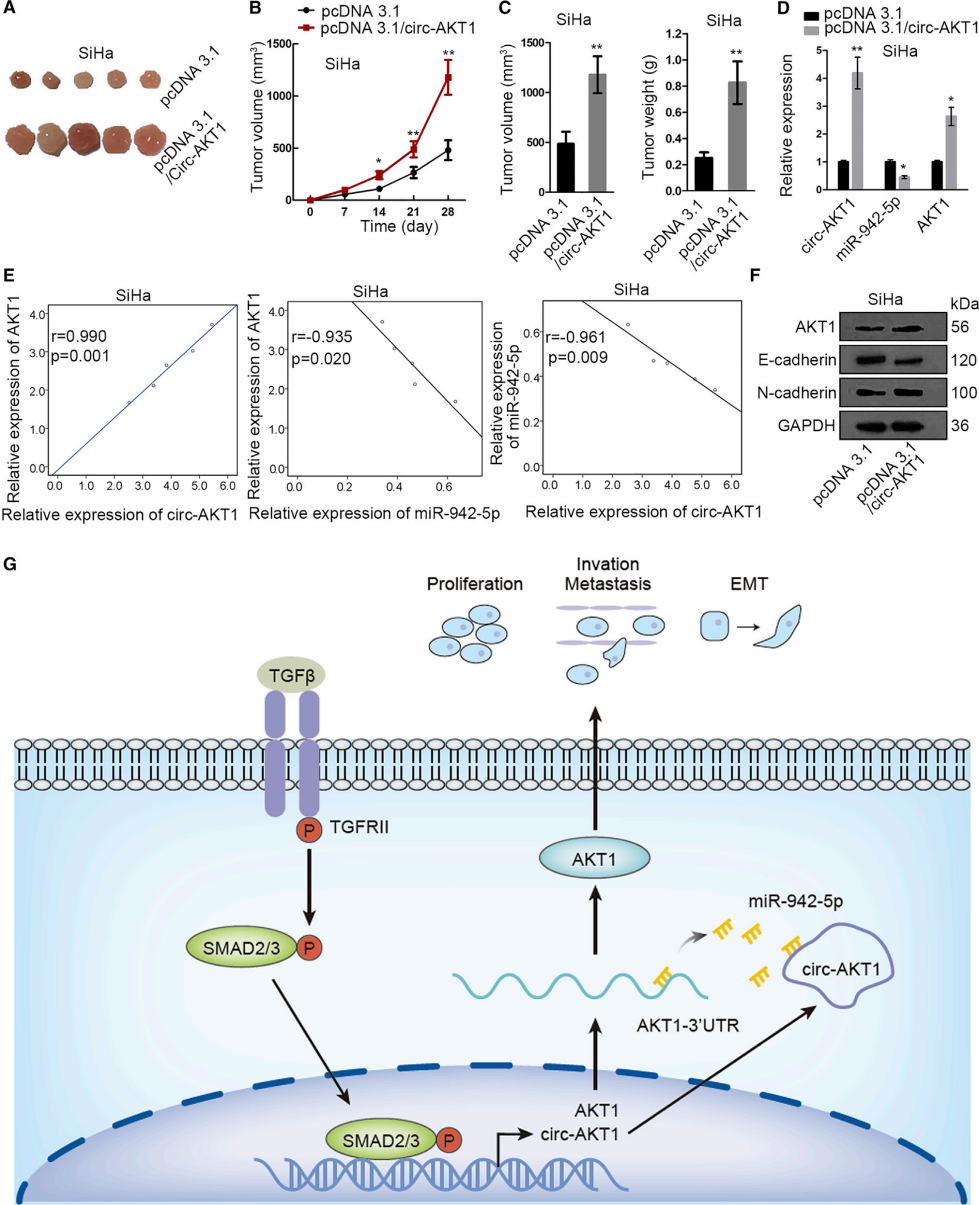
circ-AKT1 Facilitated CC Progression *In Vivo* Nude mice were injected with SiHa cells transfected with pcDNA3.1 or pcDNA3.1/circ-AKT1. (A) Pictures of xenografts in mice injected with SiHa cells transfected with pcDNA3.1 or pcDNA3.1/circ-AKT1. (B) The growth curve of xenografts in mice injected with SiHa cells transfected with pcDNA3.1 or pcDNA3.1/circ-AKT1. (C) The final tumor volume and weight in mice injected with SiHa cells transfected with pcDNA3.1 or pcDNA3.1/circ-AKT1. (D) Quantitative real-time RT-PCR measured the expressions of circ-AKT1, AKT1, and miR-942-5p in mice injected with SiHa cells transfected with pcDNA3.1 or pcDNA3.1/circ-AKT1. (E) Spearman’s correlation curve showed the correlation between circ-AKT1 and AKT1, and the correlation of miR-942-5p with circ-AKT1 and AKT1 in mice injected with SiHa cells transfected with pcDNA3.1/circ-AKT1. (F) Western blot detected the expressions of AKT1, E-cadherin, and N-cadherin in mice injected with SiHa cells transfected with pcDNA3.1 or pcDNA3.1/circ-AKT1. (G) circ-AKT1 was induced by TGF-β and could sequester miR-942-5p to upregulate AKT1 expression, so as to promote cell proliferation and invasion in CC. *p < 0.05, **p < 0.01.

Finally, we examined the effect of circ-AKT1 on the tumor growth of CC in animal models. circ-AKT1 was overexpressed in SiHa cells, and cells were injected into nude mice to generate xenografts. The tumor volume was detected over time. It turned out that injecting SiHa cells with circ-AKT1 overexpression into mice caused the generation of larger tumors compared with control (Figure 8A). Also, the tumor growth in mice was facilitated by circ-AKT1 overexpression (Figure 8B). Then we resected the tumors and validated that overexpression of circ-AKT1 could increase the volume and weight of xenografts in mice (Figure 8C). Additionally, we confirmed that expressions of circ-AKT1 and AKT1 were increased, whereas expression of miR-942-5p was reduced by circ-AKT1 overexpression *in vivo* (Figure 8D). Moreover, the positive correlation between circ-AKT1 and AKT1, and the negative correlation of miR-942-5p with circ-AKT1 and AKT1 were confirmed in the tumors from mice injected with SiHa cells/pcDNA3.1/circ-AKT1 (Figure 8E). Results of western blot assay demonstrated that the levels of AKT1 and N-cadherin were elevated, whereas the level of E-cadherin was decreased in mice injected with SiHa/pcDNA3.1/circ-AKT1 (Figure 8F). In conclusion, circ-AKT1 facilitated CC progression *in vivo*.

## Discussion

Emerging studies have shown that circRNAs are not merely splicing errors, as initially regarded,^7^ but are key regulators for the progression of cancers,^10^^,^^11^ including CC.^12^ Therefore, identification of new circRNAs participating in CC progression could provide promising treatment targets for CC patients.

Herein, we performed circRNA microarray analysis and picked the top 50 upregulated circRNAs in CC samples. Among them, AKT1 was the associated gene of circ-AKT1. Moreover, we validated that circ-AKT1 expression was elevated in CC tissues and cells, indicating the involvement of circ-AKT1 in CC. Besides, circular characteristics and cytoplasmic localization of circ-AKT1 were verified, suggesting that circ-AKT1 was a bona fide circRNA. Gain- and loss-of-function experiments suggested that circ-AKT1 promoted proliferation and invasion of CC cells.

Based on the findings that AKT1 was a host gene for circ-AKT1, we speculated the regulation of circ-AKT1 on AKT1. AKT1 is an important factor involved in the PI3K/AKT pathway,^17^^,^^18^ and it could regulate multiple cellular processes, such as proliferation, invasion, and EMT.^19^, ^20^, ^21^^,^^36^ Concordant with previous findings, AKT1 exerted oncogenic functions in CC.^22^^,^^23^ Our study also validated that AKT1 was upregulated in CC tissues and cell lines, and that silencing AKT1 inhibited cell proliferation and invasion. Additionally, it has been revealed that TGF-β is an upstream activator of AKT1,^34^^,^^35^ so we hypothesized that TGF-β was responsible for the induction of circ-AKT1 as well. Furthermore, TGF-β could induce the expression of circ-AKT1 and AKT1 in a dose-dependent manner, and that circ-AKT1 positively regulated AKT1 expression, indicating that TGF-β could stimulate AKT1 expression through circ-AKT1. Additionally, previous reports have illustrated that AKT1 could modulate EMT progress in human cancers.^36^^,^^37^ In this research, we confirmed that circ-AKT1 promoted the EMT process in CC. The above findings suggested the effects of the TGF-β/circ-AKT1/AKT1 axis on CC progression.

With regard to the molecular regulatory mechanism, circRNAs can regulate target gene expression through performing as sequesters of miRNAs in cancers,^24^^,^^25^ including in CC.^12^ In the present study, we identified two shared miRNAs for circ-AKT1 and AKT1 through bioinformatics tools, but found that only miR-942-5p was downregulated in CC cell lines and tissues. Moreover, a former study revealed that miR-942-5p was a tumor suppressor in KSHV-associated malignancies.^31^ In accordance, our findings first suggested that miR-942-5p was a tumor suppressor gene in CC. Further, the negative correlation of miR-942-5p with circ-AKT1 and AKT1 in CC samples was demonstrated. In addition, we verified the interaction of miR-942-5p with circ-AKT1 and AKT1 in CC. circ-AKT1 could modulate AKT1 expression through sponging miR-942-5p. Meanwhile, rescue assays elucidated that circ-AKT1 regulated CC progression through the miR-942-5p/AKT1 axis. Finally, *in vivo* assays validated that circ-AKT1 promoted tumor growth and regulated miR-942-5p/AKT1 expression in CC.

In conclusion, our study was the first to discover the oncogenic function of circ-AKT1 in CC, which demonstrated the mechanism that circ-AKT1 was induced by TGF-β and could sequester miR-942-5p to upregulate AKT1 expression in CC (Figure 8G). Our findings indicated circ-AKT1 as a novel promising therapeutic target for CC treatment.

## Materials and Methods

### Tissue and Serum Samples

CC tissues (n = 58) and the paired adjacent non-cancerous tissues (n = 58) were provided by the First Affiliated Hospital of Wenzhou Medical University. In addition, serum samples from CC patients (n = 58) and that from corresponding healthy volunteers (n = 40) were collected. All CC patients underwent no chemotherapy or radiotherapy prior to biopsy or surgical treatment. The collected tissue samples were maintained instantly at −80°C following resection. The permission for this study was obtained from the Ethics Committee of the First Affiliated Hospital of Wenzhou Medical University. Informed consents were signed by all patients enrolled.

### circRNA Microarray Analysis

Three pairs of CC tissues and the matched non-cancerous tissues were picked for microarray analysis. Extraction of RNA and hybridization of microarray were implemented obeying the Arraystar’s standard protocols. To be brief, RNase R (Epicenter, Madison, WI, USA) was used for RNA digestion for the removal of linear RNA and enrichment of circRNA. Thereafter, RNAs underwent amplification to generate cDNA and went through labeling by the Arraystar Human Circular RNA Array (AS-S-CR-H-V2.0) (Arraystar, Rockville, MD, USA). Eventually, the labeled RNAs were subjected to hybridization with the application of Arraystar mouse circRNA Array (v.1.0; Arraystar), and Agilent Scanner G2505C was used for the scanning.

### Cell Lines and Cell Culture

CC cell lines (HeLa, CaSki, SiHa, and C33A) and 293T cells were provided by the American Type Culture Collection (ATCC, Rockville, MD, USA), and cervical epithelial immortalized cells (H8) were provided by the Cell Resource Database of Chinese Academy of Sciences (Shanghai, China). HeLa, SiHa, C33A, and 293T cells were cultured in Dulbecco’s modified Eagle’s medium (DMEM; GIBCO-BRL; Rockville, MD, USA). CaSki and H8 cells were cultured in Roswell Park Memorial Institute (RPMI)-1640 medium (HyClone, Logan, UT, USA). All mediums were kept in an air at 37°C containing 5% CO_2_. 10% fetal bovine serum (FBS; GIBCO BRL, Grand Island, NY, USA) was used to supplement the media.

### Cell Transfection

circ-AKT1 was silenced by specific small interfering RNAs (circ-AKT1-siRNA#1, circ-AKT1-siRNA#2, and circ-AKT1-siRNA#3) and was overexpressed by pcDNA3.1 (Invitrogen, Carlsbad, CA, USA) integrated with full-length sequences of circ-AKT1. AKT1 was silenced by siRNAs targeting AKT1 (si-AKT1#1, si-AKT1#2, and si-AKT1#3). Scramble siRNAs (si-NC) and empty pcDNA3.1 vectors were negative controls (NCs). The overexpression and inhibition of miR-942-5p were realized by miR-942-5p mimics and miR-942-5p inhibitor, with NC mimics and NC inhibitor as controls (provided by Thermo Scientific, Waltham, MA, USA). The transfection of these plasmids into SiHa and CaSki cells was carried out with Lipofectamine 3000 Reagent (Thermo Fisher Scientific, CA, USA) as required, and sequences of the above-mentioned plasmids were provided in Table S1.

### Quantitative Real-Time RT-PCR

Isolation of RNAs from CC samples and cells was accomplished by using TRIzol reagent (Invitrogen, Carlsbad, CA, USA). Generation of cDNA from isolated RNAs was carried out using a reverse transcriptase kit (Takara, Tokyo, Japan) or Thermo Fisher’s K1622 kit (Thermo Fisher Scientific, USA). SYBR-Green PCR Master Mix kit (Takara, Dalian, China) was applied for quantitative real-time RT-PCR. GAPDH and U6 were the endogenous controls. Evaluation of gene expression was based on the 2^−ΔΔCt^ method. The primers for circ-AKT1, miR-942-5p, and AKT1 were obtained from Sangon Biotech (Shanghai, China). Related primer sequences were provided in Table S1.

### Circular Structure Confirmation

The circular characteristic of circ-AKT1 was examined by Sanger sequencing and PCR with the use of divergent primers and RNase R treatment. The PCR products were amplified by the divergent primers of circ-AKT1 and then integrated into the T vector, and the genomic region for circAKT1 with its flanking introns containing two inverted repeats (AluJr and AluSz) was amplified and inserted into pGEM-T vector. The Sanger sequencing was conducted by Tsingke Biotech. Then results were cross-checked by the use of the backspliced region of circ-AKT1 obtained from circBASE. Additionally, because circRNAs were normally generated from pre-mRNAs, the convergent and divergent primers were applied to amplify the linear and circular transcripts of AKT1 in both gDNA and cDNA from CC cells. Theoretically, circular transcripts of AKT1 could be amplified only by the divergent primers in cDNA rather than in gDNA. The NC was GAPDH. For the RNase R treatment, RNAs extracted from CC cells were incubated for 45 min at 37°C with the addition of 10 U RNase R (20 U/μL; Epicenter, Madison, WI, USA) in 10 μL volume, followed by the incubation at 70°C for 10 min for the deactivation of RNase R. Thereafter, the treated RNAs were analyzed by quantitative real-time RT-PCR.

### Northern Blot Assay

Northern blotting was carried out by utilizing the DIG Northern Starter Kit (Roche Diagnostics). The probed membrane was washed two times by using 2× SSC (300 mM NaCl, 30 mM sodium citrate [pH 7.0]) supplemented with 0.1% sodium dodecyl sulfate (SDS) for 6 min at room temperature and another two times with 0.1× SSC containing 0.1% SDS for 16 min at 69°C. Horseradish peroxidase (HRP)-conjugated anti-digoxigenin was applied to measure the hybridized probe by chemiluminescent imaging with a lumino-image analyzer (LAS-4000; GE Health Care, Hino, Tokyo, Japan), and GAPDH was the NC.

### CCK-8 Assay

CC cell proliferation was evaluated by CCK-8 (Dojindo, Japan). In brief, 1 × 10^3^ CC cells (SiHa and CaSki) were plated into each well of 96-well plates, and CCK-8 solution (10 μL) was added to each well after cells were incubated for 0, 24, 48, 72, and 96 h, followed by the incubation of 2 h. The absorbance was evaluated at 450 nm using a spectrophotometer.

### EdU Assay

EdU assay was implemented with a Cell-Light EdU DNA Cell Proliferation Kit (RiboBio, Guangzhou, China). After being incubated with EdU for 3 h, the cells were fixed and went through nuclei staining with 4,6-diamidino-2-phenylindole (DAPI; Sigma-Aldrich, St. Louis, MO, USA). Images were captured with the inverted fluorescence microscope (Carl Zeiss, Jena, Germany). The ratio of EdU-positive cells was evaluated.

### Colony Formation Assay

Cells were seeded into the six-well plates (5 × 10^2^ cells/well) and underwent incubation for 14 days. Thereafter, cells were fixed in methanol and subjected to crystal violet staining. The colonies consisting of over 50 cells were counted under a microscope.

### Transwell Invasion Assay

Cell invasion was examined by utilizing Corning Polycarbonate Membrane Insertin transwell chamber (Corning Costar, Cambridge, MA, USA). After 24 h of transfection, CC cells were placed in the upper compartment pre-coated with Matrigel (Sigma-Aldrich, St. Louis, MO, USA) in non-serum medium. 20% FBS was used to supplement the medium in the lower compartment as a chemoattractant. After incubation for 2 days, the cells that had not invaded were removed by cotton swabs. Cells invading to the bottom of the membrane were fixed and underwent staining using 0.1% crystal violet. The pictures were captured utilizing the microscope. The number of cells was evaluated by ImageJ software.

### FISH Assay

After the culturing on coverslips, fixation, and permeabilization, the coverslips went through hybridization overnight utilizing hybridization buffer (Geneseed Biotech, Guangzhou, China) with the addition of digoxin as well as biotin-labeled single-stranded DNA probes at 37°C. Digoxin-labeled probes for circ-AKT1 were obtained from GenePharma (Shanghai, China). Detection of signals was conducted by using Cy3-conjugated anti-digoxin, as well as fluorescein isothiocyanate (FITC)-conjugated anti-biotin antibodies (Jackson ImmunoResearch, West Grove, PA, USA). Then cell nuclei underwent counterstaining by utilizing DAPI. Images were captured by utilizing Zeiss LSM 700 confocal microscope (Carl Zeiss, Oberkochen, Germany).

### IF Staining Assay

After the fixation in 4% formaldehyde solution and the wash in PBS, CC cells were lysed utilizing 0.5% Triton-X. Then cells were subjected to incubation utilizing 5% BSA for 5 min to prevent non-specific background staining. Thereafter, cells underwent incubation with the primary antibodies against E-cadherin and N-cadherin for 2 h at 37°C, followed by incubation for 1 h with the secondary antibody. Later, cells were subjected to the treatment of DAPI for 10 min. The fluorescence microscope was used to observe the cells.

### RNA Pull-Down Assay

In brief, transcripts of circ-AKT1 were transcribed utilizing T7 RNA polymerase (Ambion Life, Taoyuan, Taiwan). The purified RNAs were biotin labeled by Biotin RNA Labeling Mix (Ambion Life). The cell lysates^39^ were incubated with the biotin-labeled or non-biotin-labeled probes of circ-AKT1. Streptavidin magnetic beads were then added to prepare the probe-magnetic bead complexes. After washing the bead complexes, the eluted RNAs were examined by quantitative real-time RT-PCR.

### Subcellular Fractionation

Separation of cytoplasmic and nuclear RNA in SiHa and CaSki cells was carried out using Cytoplasmic and Nuclear RNA Purification Kit (Norgenbiotek Corporation, Thorold, ON, Canada). The relative expression level of circ-AKT1 was subjected to quantitative real-time RT-PCR. U6 (nucleus control) and GAPDH (cytoplasm control) were employed for normalization.

### RIP

The RIP assay was implemented with the application of Magna RIP RNA Binding Protein Immunoprecipitation Kit (Bersinbio, Guangzhou, China). After being lysed in the complete RIP lysis buffer, cell lysates were obtained and separated into two equal parts for the overnight incubation with antibody against anti-Argonaute2 (Ago2) (Millipore, Billerica, MA, USA) or IgG (Millipore) with rotation at 4°C. Then the cell lysates were incubated for 1 h with the addition of magnetic beads. Thereafter, Proteinase K was added and samples underwent 1-h incubation. The expressions of purified RNAs were determined by quantitative real-time RT-PCR analysis.

### Dual-Luciferase Reporter Assay

The 3′ UTR regions of AKT1 and circ-AKT1 containing binding sequences for miR-942-5p were generated into the pmirGLO luciferase vectors (Promega, Madison WI, USA) to generate circ-AKT1-WT and AKT1-WT reporters, and the circ-AKT1-MUT and AKT1-MUT reporters (with the MUT type of sequences) were generated as well. The circ-AKT1-WT, circ-AKT1-MUT, AKT1-WT, or AKT1-MUT was respectively transfected with miR-942-5p mimics or NC mimics into 293T cells using Lipofectamine 2000 (Invitrogen). Following transfection for 2 days, the luciferase activities were determined by the application of dual-luciferase reporter assay kit (Promega, Madison WI, USA), and relative luciferase activities were normalized to the Renilla luciferase activities.

### Western Blot

Extracts of total proteins were obtained from cells with the use of radioimmunoprecipitation assay (RIPA) lysis buffer (Beyotime, Shanghai, China) with the supplementation of 1% PMSF (Roche, Basel, Switzerland). After the separation of equal quantity of protein samples by 10% SDS polyacrylamide gel electrophoresis (SDS-PAGE) gels, the proteins were shifted to polyvinylidene fluoride (PVDF) membranes, which were later sealed with skim milk. Blots were subjected to 12-h incubation with primary antibodies and then 1-h incubation with secondary antibody. Electrochemiluminescence (ECL) kit (Tanon) was applied for the visualization of protein signals. GAPDH was the internal control. Primary antibodies were anti-E-cadherin, anti-N-cadherin, anti-AKT1, anti-PCNA, and anti-Ki67 from Abcam (Cambridge, UK).

### Xenograft Assay

The male BALB/c nude mice that were 4 weeks old were kept under non-pathogen conditions. The animal study procedures had gained the approval of the Animal Care Committee of The First Affiliated Hospital of Wenzhou Medical University. SiHa cells transfected with pcDNA3.1 or pcDNA3.1/circ-AKT1 were subcutaneously injected into either side of the flank area of the nude mice (n = 5 in each group) to generate the xenografts. The tumor growth was determined 7 days at a time. Tumor volume was evaluated based on the following equation: length × width^2^ × 0.5. Twenty-eight days after injection, mice underwent euthanasia, and tumors were dissected for further assays.

### Bioinformatics Tools

A total of 107 putative miRNAs targeting AKT1 were obtained from PITA (https://genie.weizmann.ac.il/pubs/mir07/mir07_data.html), and 20 miRNAs targeted by circ-AKT1 were provided by CircInteractome (http://circinteractome.nia.nih.gov/).

### Statistical Analysis

Expression of quantitative data was based on mean ± standard deviation. Analysis of statistics was carried out on GraphPad Prism v.5.01 (GraphPad, La Jolla, CA, USA) software. Analysis of area under the ROC curve (AUC) was used to estimate the diagnostic significance of circ-AKT1. All experiments were performed at least three times. Student’s t test (two-tailed) and one-way ANOVA were employed for comparing the differences between two groups or the differences among multiple groups. The statistical significance of differences was determined by p < 0.05.

## Author Contributions

R.O. and L.M. conceived and designed the study. H.T. and S.L. performed the experiments. H.Z. and L.Z. assisted with analyzing the data. R.O. drafted the manuscript, which was reviewed by Y.R. and Y.X.

## Conflicts of Interest

The authors declare no competing interests.

## Supplemental Information

Document S1. Figures S1–S3 and Tables S1 and S2

Document S2. Article plus Supplemental Information
