# Supplementary material for: circRNA-AKT1 Sequesters miR-942-5p to Upregulate AKT1 and Promote Cervical Cancer Progression
Source: Mol Ther Nucleic Acids. 2020 Jan 16;20:308–22. doi: 10.1016/j.omtn.2020.01.003 (PMC7078494; doi:10.1016/j.omtn.2020.01.003)
Supplement: Document S1. Figures S1–S3 and Tables S1 and S2 [file mmc1.pdf]

OMTN, Volume *20*

Supplemental Information

circRNA-AKT1 Sequesters miR-942-5p to Upregulate AKT1 and Promote Cervical Cancer Progression

Rongying Ou, Laiming Mo, Huijing Tang, Shaolong Leng, Haiyan Zhu, Liang Zhao, Yi Ren, and Yunsheng Xu

Supplementary figure 1 Heat-map exhibited the expression of circRNAs in CC tissues and the matched adjacent non-cancerous tissues. (A-B) The heat-map displayed the expression of related circRNAs in CC tissues (n = 3) and the matched adjacent non-cancerous tissues (n = 3).

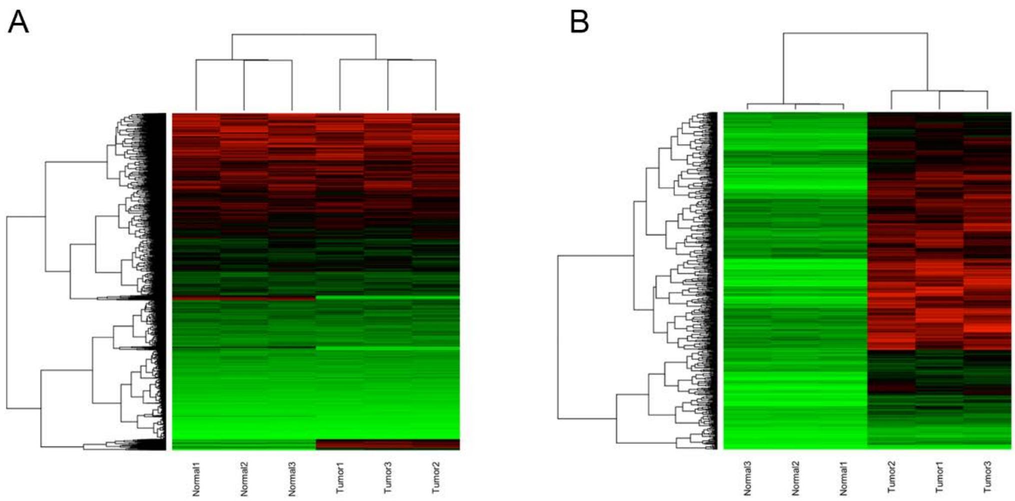

Supplementary figure 2 Northern blot measured the expression of AKT1 or circ-AKT1 in differently treated groups. (A) Northern blot assay detected the RNA expression of AKT1 and circ-AKT1 in RNase R treatment. (B) Northern blot assay detected the RNA expression of AKT1 with or without flanking intron treatment (Alu). GAPDH was negative control.

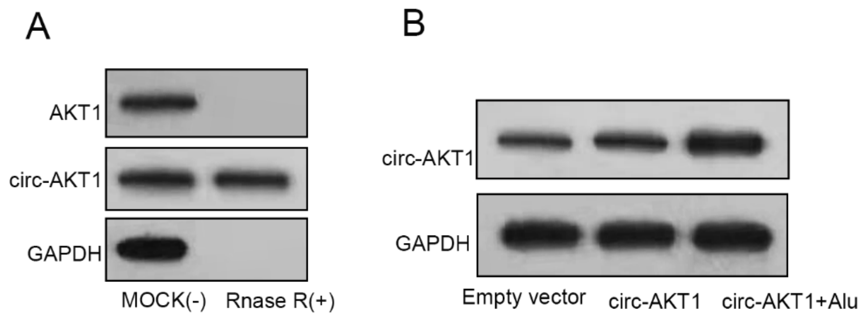

Supplementary figure 3 The expression of TGF-β in CC tissues and cells was examined. (A-B) RT-qPCR detected that the expression of TGF-β in CC tissues (n = 58) and cells as well as in corresponding control groups (n = 58). $^{**}P < 0.01$

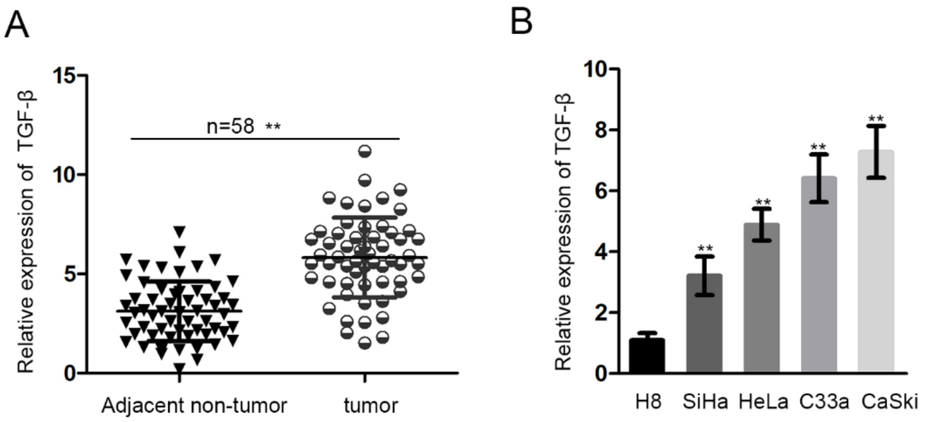

Table legends

Supplementary Table 1 Related sequences of genes were provided. Related PCR primer sequences and interference/overexpression sequences were exhibited.

Gene name:	PCR primer sequences:	Covergent primers for circ-AKT:	
circ-AKT1	F：CGTTCTTCTCCGAGTGCAG<br>R：ACGTTCTTCTCCGAGTGCAG	F：TGTTGAGGGTTGTCTCCGTG<br>R：GGACAGTCATGAGCTTCGCT	
miR-942-5p	F：CUUCUCUGUUUUGGCCAUGUG<br>R：CTCTACAGCTATATTGCCAGCCAC		
AKT1	F: CCTTCTTGAGCAGCCCTGAA<br>R: TACGAGATGATGTGCGGTCG		
**Plamids name:**	**Sequences:**		
siRNA	CUGACCAAGGUGGAAGAACAG		
circ-AKT1-siRNA#1	GUGGAAGAACAGCUUGGUCAG		
circ-AKT1-siRNA#2	CACCACCUGACCAAGCUGUUC		
circ-AKT1-siRNA#3	CACCUGACCAAGCUGUUCUUC		
si-AKT1#1	GCACCUUCAUUGGCUACAA		
si-AKT1#2	GGCCCAACACCUUCAUCAU		
si-AKT1#3	GGAGACUGACACCAGGUAU		
NC inhibitor	CACAUGGCCAAAACCUUCUCUA		
miR-942-5p inhibitor	CACAUGGCCAAAACAGAGAAGA		
NC mimics	UAGAGAAGGUUUUGGCCAUGUG		
miR-942-5p mimics	UCUUCUCUGUUUUGGCCAUGUG		

Supplementary Table 2 The accession number for the first 50 upregulated circRNAs in CC tissues was provided.

**circRNA**	**Accession number**				
circ-AKT1	NM_005163				
circ-DCLK2	NR_036614				
circ-CAMK2D	NM_001221				
circ-PLOD2	NM_000935				
circ-AGGF1	NM_018046				
circ-BBS9	NM_014451				
circ-EPB41	NM_004437				
circ-ANKS1B	NM_152788				
circ-TRIAP1	NM_016399				
circ-ANKRD36	NM_001164315				
circ-MBOAT2	NM_138799				
circ-KDM4B	NM_015015				
circ-HSF4	NM_001040667				
circ-EXOC6	NM_019053				
circ-PITPNB	NM_012399				
circ-PTPN12	NM_002835				
circ-PARD3	NM_019619				
circ-FGD5	NM_152536				
circ-GLB1	NM_000404				
circ-PTDSS1	NM_014754				
circ-PHACTR4	NM_023923				
circ-RAPGEF1	NM_005312				
circ-ZNF717	NM_001128223				
circ-PAN3	NM_175854				
circ-SLC4A5	NM_021196				
circ-RNPS1	NM_006711				
circ-HDAC11	NM_024827				
circ-PAPPA	NM_002581				
circ-EPB41L5	NM_020909				
circ-VCAN	NM_004385				
circ-PHKB	NM_000293				
circ-WSB1	NM_015626				
circ-TRIM37	NM_015294				
circ-DPY19L1	NM_015283				
circ-MAST2	NM_015112				
circ-AK127472	uc003qaq.1				
circ-NFAT5	NM_006599				
circ-SLMAP	NM_007159				
circ-ADAMTS6	NM_197941				
circ-MLLT6	NM_005937				
circ-NR3C2	NM_000901				
circ-KMT2E	NM_018682				
circ-SLC3A2	NM_002394				
circ-DOCK2	NM_004946				
circ-STXBP4	NM_178509				
circ-CANX	NM_001746				
circ-TRIO	NM_007118				
circ-FADS2	NM_004265				
circ-STARD7	NM_020151				
circ-NAV3	NM_014903				

1. Over-expressed loop-assisted sequence of circ-AKT1：

pcDNA3.1/ circ-AKT1

AGTAGAGACGGGGTTTCACCATGTTGGCCAGGCTGGTCTTCACTTTTTGTAAAGGTACGTACTAATGACTTTTTTTTTATACT
TCAGCTGTTCTTCCACCTGTCCCGGGAGCGTGTGTTCTCCGAGGACCGGGCCCGCTTCTATGGCGCTGAGATTGTGTCAGCCC
TGGACTACCTGCACTCGGAGAAGAACGTGGTGTACCGGGACCTCAAGGTGCGCTGGCGGGCAGGCAGGGGGGCAGGGCCCT
GGGGGCCTGGCGGCACTGACCTGAGGCCACCTTTCCCCTAGCTGGAGAACCTCATGCTGGACAAGGACGGGCACATTAAG
ATCACAGACTTCGGGCTGTGCAAGGAGGGGATCAAGGACGGTGCCACCATGAAGACCTTTTGCGGCACACCTGAGTACCT
GGCCCCCGAGGTGTGCGCCCCACCTGCGTGCATACGCGTTGCTGCGTCCCCACGTCCTGAGCACACGCAATGCTGTGTCC
TCTCTGTGCCCCAAGCACGTCACACCTCCCCCGGCAGTGTCCCGGACACCCCTTGATGCCGAGTCCTGCCCATCTGCCAC
CCGTGCAGGTGCTGGAGGACAATGACTACGGCCGTGCAGTGGACTGGTGGGGGCTGGGCGTGGTCATGTACGAGATGATG
TGCGGTCGCCTGCCCTTCTACAACCAGGACCATGAGAAGCTTTTTGAGCTCATCCTCATGGAGGAGATCCGCTTCCCGCG
CACGCTTGGTCCCGAGGCCAAGTCCTTGCTTTCAGGGCTGCTCAAGAAGGACCCCAAGCAGAGGTGAGGGCCGCCCATCC
CAGCTACAGGCTACACCTCCATCCCCTCATCCCCAGGCTGCACCTGCCCCTGCGCCAGGCGGTCCTGGCACCTCCCAGAC
TACACTGATAGCCAAAGCTTGATGTCCTTGGCCAGGGCTGATCTCCAAAGCCTCAGGCCCAAGCAGAGTGGCGGGCAGGC
CGGGGTCAGTTGGGCCTCTGTCCCCAACCCTGCAGCCTGCACCCACCCACTCAGGAAGCCCCTGCCCTGCCGTGAGCTCT
GTGGTGCTTTGCTACCCACAGCTGCTCAGGGACGCTGCACCACCGGCTCCCCTCCCTGGCCCCGGAACGTCCTGTCTGGC
GGGCCCTACATCACAGGAGGAAGGGGCCTGAACCCAGGGCCTGGGCAGGTGGCGGTACCGACACTGTGGCCTTGTTTCCT
GCCTGCAGGCTTGGCGGGGGCTCCGAGGACGCCAAGGAGATCATGCAGCATCGCTTCTTTGCCGGTATCGTGTGGCAGCA
CGTGTACGAGAAGAAGGTGCGGCTGCTCCCCGCATATTCACGCGCACGCATGCTCCCCACATATCCACACTCACGCATGC
ACGTGGCACGCTCGCCAGATTTCCCACACACTCGCCCTCACCTCAGGAGCCTGCTGCAGTCCTGGTACAAGGAGGGCCTT
GCTGCACCAACCTCAGCGCCTGGTGCTCAGAGGCTCTGGCACTGCCGGGTTCCACCAGGAAACTGGCCTGGTCCTCATTT
CCTCCTCCCCTCGGAGGTGTGTCACACTCTGAGTTTCCTCCTCCCCTCGGAGGTGTGTCACACTCTGAGTGCCAGCCTTG
GGGTTCCCTTCCCTGACGCTGTGCAGTGAAGGCTGGCTGGTGGTGGACCAGGGGTGCTGCCCCTTGGCCTCCACGAGTTC
CTCCTGTTTGACCTCAATCCCTTCTGCCGCAAGGAGAGCCCAGCCCTATTTCTGGCTGTGCAGGGACAGGGACAGCACCT
ACTTTTCTGGCACATGGGGGAGTCCGCCCTGGAGGGAGGCAGCTCTTTCTGCATGAGTCGCCATCCTGGGTGCTCATCTT
TCAGGGACCCTAGGAGCCCTGGCCATCACCCCAGCTGATGGGGTCTGCCAGCTGGGTTCGGAAGCCTGCACTCTGAGATG
CTGGGTGCCCTGTCACCAGGTGTGCTTCTGCCCCGTTCCCAGCCTGCCCCTCCCCTGAATGCCTGTCCTAGGCTGTGTGC
AGCTGGTCGGGCTGGGCAGCCCTGGGACCCAGCTGGGCACCTGTTCTCCCATTAACACAGGGTGCCCTGGTCCTGGTCCC
CGTTCCTGCCTTGAGGCCTGCCACCCTCCTGCCCTGCCCTCCAAGGGTATGGGGGGCCTGGGCAGTGCTCAACCTCTGCC
CAGCCTTGACCAGAGACCTTGCTAATTGACAATGGACAATGCTGGTGCCCAGCCCAGACTCTGGGGTGGGGCAGTGCTTG
AGGGGGTCCTTGACTGTGGGACCTGAGGGGCTCTCTGGCAGCTCCTCACGTGTGCACATCACCTTATAGTCACCCTTCAT
CCTGGGTCATTGAGAGTCCTGTCTGCAGCCAGATGCCAGCAGGCTGTGGTCCTGTAGTCCCTGAGCCATGGAGGCAGGGT
GGGCTCATCCTGTGCACCTTGTTCCACAGTCTCCCTCTTTCCCAATTTACAAGAAGACCAATAAGAAAAATAAGAAAACA
AGAAAGAAAAAGGAAAAATAAAGTTATCACCTCCTGGTAGCAGGGAGGGTCTCTCAGAGCTGGGCTGCACTACCTCCTGT
CGCCTGCTGGGGCTGCTGTGAGGGCAGCTTTGCGTCTCAGCTGTTGAGGGTTGTCTCCGTGCTCCTGCACCCCTCATGTC
CCTCCCTCCCAGAGCTGCCTCTGTGGGGGGCTGGTGGCTTGCTGCTCTCTGACATCAGTCCTGCCTGGAGACCCCTTGGA
GATCCAGGTGCTTTGAAGGTCTTGAGCACACTTGAGGGTGTGCTGGGAGTGGGGAGCGAAGCTCATGACTGTCCCGTCTG
CCCACCTCTGCAGCTCAGCCCACCCTTCAAGCCCCAGGTCACGTCGGAGACTGACACCAGGTATTTTGATGAGGAGTTCA
CGGCCCAGATGATCACCATCACACCACCTGACCAAGGTAAGAAGCAAGGAAAAGAATTAGAGACCAGCCTGGCCAACATGGTG
AAACCTTGTCTCTACT

2. Hsa_circ_0033550 circRNA Genomic Sequence:

CTGTTCTTCCACCTGTCCCGGGAGCGTGTGTTCTCCGAGGACCGGGCCCGCTTCTATGGCGCTGAGATTGTGTCAGCCCT
GGACTACCTGCACTCGGAGAAGAACGTGGTGTACCGGGACCTCAAGGTGCGCTGGCGGGCAGGCAGGGGGGCAGGGCCCT
GGGGGCCTGGCGGCACTGACCTGAGGCCACCTTTCCCCTAGCTGGAGAACCTCATGCTGGACAAGGACGGGCACATTAAG
ATCACAGACTTCGGGCTGTGCAAGGAGGGGATCAAGGACGGTGCCACCATGAAGACCTTTTGCGGCACACCTGAGTACCT
GGCCCCCGAGGTGTGCGCCCCACCTGCGTGCATACGCGTTGCTGCGTCCCCACGTCCTGAGCACACGCAATGCTGTGTCC
TCTCTGTGCCCCAAGCACGTCACACCTCCCCCGGCAGTGTCCCGGACACCCCTTGATGCCGAGTCCTGCCCATCTGCCAC
CCGTGCAGGTGCTGGAGGACAATGACTACGGCCGTGCAGTGGACTGGTGGGGGCTGGGCGTGGTCATGTACGAGATGATG
TGCGGTCGCCTGCCCTTCTACAACCAGGACCATGAGAAGCTTTTTGAGCTCATCCTCATGGAGGAGATCCGCTTCCCGCG
CACGCTTGGTCCCGAGGCCAAGTCCTTGCTTTCAGGGCTGCTCAAGAAGGACCCCAAGCAGAGGTGAGGGCCGCCCATCC
CAGCTACAGGCTACACCTCCATCCCCTCATCCCCAGGCTGCACCTGCCCCTGCGCCAGGCGGTCCTGGCACCTCCCAGAC
TACACTGATAGCCAAAGCTTGATGTCCTTGGCCAGGGCTGATCTCCAAAGCCTCAGGCCCAAGCAGAGTGGCGGGCAGGC
CGGGGTCAGTTGGGCCTCTGTCCCCAACCCTGCAGCCTGCACCCACCCACTCAGGAAGCCCCTGCCCTGCCGTGAGCTCT
GTGGTGCTTTGCTACCCACAGCTGCTCAGGGACGCTGCACCACCGGCTCCCCTCCCTGGCCCCGGAACGTCCTGTCTGGC
GGGCCCTACATCACAGGAGGAAGGGGCCTGAACCCAGGGCCTGGGCAGGTGGCGGTACCGACACTGTGGCCTTGTTTCCT
GCCTGCAGGCTTGGCGGGGGCTCCGAGGACGCCAAGGAGATCATGCAGCATCGCTTCTTTGCCGGTATCGTGTGGCAGCA
CGTGTACGAGAAGAAGGTGCGGCTGCTCCCCGCATATTCACGCGCACGCATGCTCCCCACATATCCACACTCACGCATGC
ACGTGGCACGCTCGCCAGATTTCCCACACACTCGCCCTCACCTCAGGAGCCTGCTGCAGTCCTGGTACAAGGAGGGCCTT
GCTGCACCAACCTCAGCGCCTGGTGCTCAGAGGCTCTGGCACTGCCGGGTTCCACCAGGAAACTGGCCTGGTCCTCATTT
CCTCCTCCCCTCGGAGGTGTGTCACACTCTGAGTTTCCTCCTCCCCTCGGAGGTGTGTCACACTCTGAGTGCCAGCCTTG
GGGTTCCCTTCCCTGACGCTGTGCAGTGAAGGCTGGCTGGTGGTGGACCAGGGGTGCTGCCCCTTGGCCTCCACGAGTTC
CTCCTGTTTGACCTCAATCCCTTCTGCCGCAAGGAGAGCCCAGCCCTATTTCTGGCTGTGCAGGGACAGGGACAGCACCT
ACTTTTCTGGCACATGGGGGAGTCCGCCCTGGAGGGAGGCAGCTCTTTCTGCATGAGTCGCCATCCTGGGTGCTCATCTT
TCAGGGACCCTAGGAGCCCTGGCCATCACCCCAGCTGATGGGGTCTGCCAGCTGGGTTCGGAAGCCTGCACTCTGAGATG
CTGGGTGCCCTGTCACCAGGTGTGCTTCTGCCCCGTTCCCAGCCTGCCCCTCCCCTGAATGCCTGTCCTAGGCTGTGTGC
AGCTGGTCGGGCTGGGCAGCCCTGGGACCCAGCTGGGCACCTGTTCTCCCATTAACACAGGGTGCCCTGGTCCTGGTCCC
CGTTCCTGCCTTGAGGCCTGCCACCCTCCTGCCCTGCCCTCCAAGGGTATGGGGGGCCTGGGCAGTGCTCAACCTCTGCC
CAGCCTTGACCAGAGACCTTGCTAATTGACAATGGACAATGCTGGTGCCCAGCCCAGACTCTGGGGTGGGGCAGTGCTTG
AGGGGGTCCTTGACTGTGGGACCTGAGGGGCTCTCTGGCAGCTCCTCACGTGTGCACATCACCTTATAGTCACCCTTCAT
CCTGGGTCATTGAGAGTCCTGTCTGCAGCCAGATGCCAGCAGGCTGTGGTCCTGTAGTCCCTGAGCCATGGAGGCAGGGT
GGGCTCATCCTGTGCACCTTGTTCCACAGTCTCCCTCTTTCCCAATTTACAAGAAGACCAATAAGAAAAATAAGAAAACA
AGAAAGAAAAAGGAAAAATAAAGTTATCACCTCCTGGTAGCAGGGAGGGTCTCTCAGAGCTGGGCTGCACTACCTCCTGT
CGCCTGCTGGGGCTGCTGTGAGGGCAGCTTTGCGTCTCAGCTGTTGAGGGTTGTCTCCGTGCTCCTGCACCCCTCATGTC
CCTCCCTCCCAGAGCTGCCTCTGTGGGGGGCTGGTGGCTTGCTGCTCTCTGACATCAGTCCTGCCTGGAGACCCCTTGGA
GATCCAGGTGCTTTGAAGGTCTTGAGCACACTTGAGGGTGTGCTGGGAGTGGGGAGCGAAGCTCATGACTGTCCCGTCTG
CCCACCTCTGCAGCTCAGCCCACCCTTCAAGCCCCAGGTCACGTCGGAGACTGACACCAGGTATTTTGATGAGGAGTTCA
CGGCCCAGATGATCACCATCACACCACCTGACCAAG
