# Supplementary material for: circRNA-AKT1 Sequesters miR-942-5p to Upregulate AKT1 and Promote Cervical Cancer Progression
Source: Mol Ther Nucleic Acids. 2020 Jan 16;20:308–22. doi: 10.1016/j.omtn.2020.01.003 (PMC7078494; doi:10.1016/j.omtn.2020.01.003)
Supplement: Document S2. Article plus Supplemental Information [file mmc2.pdf]

Molecular Therapy
Nucleic Acids
Original Article

circRNA-AKT1 Sequesters miR-942-5p to Upregulate AKT1 and Promote Cervical Cancer Progression

Rongying Ou,[1,2] Laiming Mo,[3] Huijing Tang,[4] Shaolong Leng,[4] Haiyan Zhu,[2] Liang Zhao,[1] Yi Ren,[5] and Yunsheng Xu[1,4]

[1]Laboratory for Advanced Interdisciplinary Research, Institutes of Translational Medicine, The First Affiliated Hospital of Wenzhou Medical University, Wenzhou, China; [2]Department of Gynaecology and Obstetrics, The First Affiliated Hospital of Wenzhou Medical University, Wenzhou, China; [3]Clinical Laboratory, The Seventh Affiliated Hospital, Sun Yat-sen University, Shenzhen, China; [4]Department of Dermatovenereology, The Seventh Affiliated Hospital, Sun Yat-sen University, Shenzhen, China; [5]Department of Biomedical Sciences, Florida State University College of Medicine, Tallahassee, FL, USA

Statistics show that the prognosis of cervical cancer (CC) is poor, and the death rate of CC in advanced stage has been rising in recent years. Increasing evidence has demonstrated that circular RNAs (circRNAs) serve as promising biomarkers in human cancers, including CC. The present study planned to find out the circRNA involved in CC and to explore its regulatory mechanism in CC. We discovered the new circRNA, circ-0033550, upregulated in CC. Its associated gene was AKT (also known as protein kinase B) serine/threonine kinase 1 (AKT1), so we renamed circ-0033550 as circ-AKT1. We confirmed the high expression of circ-AKT1 in CC samples and cell lines, as well as the circle structure of circ-AKT1. Functionally, gain- and loss-of-function experiments indicated that circ-AKT1 and AKT1 promoted CC cell proliferation and invasion. Moreover, circ-AKT1 and AKT1 were induced by transforming growth factor beta (TGF-β) and facilitated EMT (epithelial-mesenchymal transition) in CC. Mechanically, we illustrated that circ-AKT1 upregulated AKT1 by sponging miR-942-5p. Rescue assays confirmed the role of the circ-AKT1/miR-942-5p/AKT1 axis in CC progression. *In vivo* assays validated that circ-AKT1 promoted tumor growth in CC. Overall, circRNA-AKT1 sequestered miR-942-5p to upregulate AKT1 and promote CC progression, which may offer a new molecular target for the treatment improvement of CC.

INTRODUCTION

Cervical cancer (CC) is a class of cancer in the gynecologic system, taking up a large proportion of global cancer-associated mortality.[1,2] Despite that the past three decades have witnessed a decrease in the incidence and death rate of CC owing to the wide application of Pap smear screening tests, CC patients at advanced stage remain suffering from a dismal prognosis.[3,4] Therefore, deep investigation on the mechanism behind CC progression is beneficial to the improvement of CC treatment and prognosis.

Circular RNAs (circRNAs) are a subset of non-coding RNAs (ncRNAs) incapable of translating proteins.[5,6] Initially referred to as splicing errors,[7] circRNAs have a structure of covalently closed loop short of 3′ and 5′ ends.[8,9] However, more and more studies have demonstrated the implication of circRNAs with aberrant expression in the progression of cancers,[10,11] including in CC.[12] circRNAs have been reported to affect tumor-related cellular activities, such as cell proliferation, invasion, as well as epithelial-mesenchymal transition (EMT) process.[13–16] circRNA-0033550 is a newly found circRNA by our study. It aroused our interest not only because of its upregulated expression in CC samples discovered by microarray, but also because of the associated relation between circRNA-0033550 and AKT (protein kinase B) serine/threonine kinase 1 (AKT1) gene. Therefore, we renamed circRNA-0033550 as circ-AKT1 and focused on exploring its role in CC.

AKT1, a serine/threonine kinase with high conservative property, is a pivotal component involved in the phosphatidylinositol 3-kinase (PI3K)/AKT pathway.[17,18] Moreover, AKT1 is responsible for several cellular processes, such as proliferation, migration, and invasion of cells.[19–21] Additionally, the involvement of AKT1 in CC has been demonstrated by several studies.[22,23] However, the correlation of AKT1 with circ-AKT1 has never been investigated.

Mechanistic research has demonstrated that circRNAs can work through sponging microRNAs (miRNAs) and regulating downstream gene expression so as to regulate human cancers,[24,25] including CC.[12] miRNAs, defined as small ncRNAs with 19–24 nt, are proved to be essential modulators of pathogenesis and development of numerous cancers,[26] including CC.[27,28] Via targeting the 3′ untranslated region (3′ UTR) of messenger RNAs (mRNAs) to cause translation repression or degradation, miRNAs can silence the expressions of target

Received 26 April 2019; accepted 3 January 2020;
https://doi.org/10.1016/j.omtn.2020.01.003.

Correspondence: Yunsheng Xu, Laboratory for Advanced Interdisciplinary Research, Institutes of Translational Medicine, The First Affiliated Hospital of Wenzhou Medical University, Wenzhou 325000, China.
E-mail: shengqix730369@163.com

This is an open access article under the CC BY license (http://creativecommons.org/licenses/by/4.0/).

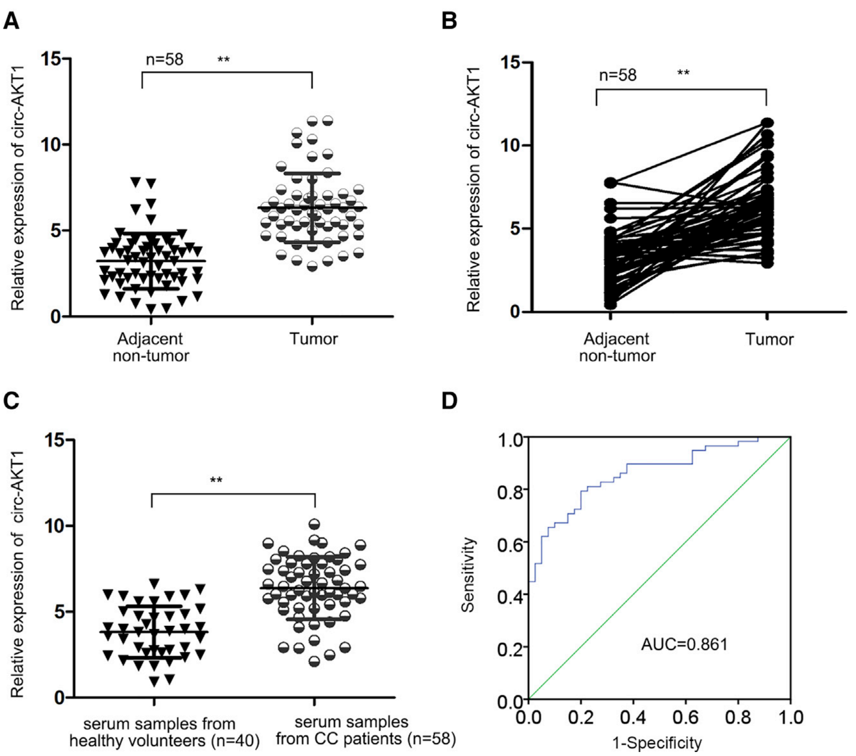

Figure 1. circ-AKT1 Was Upregulated in CC

(A and B) Quantitative real-time RT-PCR detected the relative expression of circ-AKT1 in 58 CC tissues and 58 matched non-cancerous tissues. (C) Quantitative real-time RT-PCR measured the relative expression of circ-AKT1 in the serum of CC patients (n = 58) and in that of healthy volunteers (n = 40). (D) ROC curve displayed the diagnostic performance of circ-AKT1 for CC patients. **p < 0.01.

mRNAs.[29,30] miR-942-5p has been reported to exert suppressive function in Kaposi's sarcoma-associated herpesvirus (KSHV)-associated malignancies.[31] However, its function has never been explored in CC, and its relation with circ-AKT1 and AKT1 remains elusive.

The purpose of our study is to find out the role and mechanism of circ-AKT in CC progression, and we conclude that circRNA-AKT1 sequesters miR-942-5p to upregulate AKT1 and promote CC progression.

RESULTS

circ-AKT1 Was Upregulated in CC

First, we identified that circRNAs participated in CC through circRNA microarray. As shown by the heatmap (Figures S1A and S1B), 1,842 circRNAs were upregulated and 1,620 circRNAs were downregulated in CC tissues (n = 3) compared with the matched adjacent non-cancerous tissues (n = 3), and the top 50 upregulated circRNAs were picked up for further research (Table S2). From the 50 upregulated circRNAs, we selected circ-AKT1, whose annotated gene was AKT1. Besides, AKT1 has been widely reported to be a frequently activated kinase in cancers,[23,32,33] exerting important functions in regulating cellular activities such as cell proliferation and invasion.[19–21] We then confirmed that circ-AKT1 was highly expressed in CC samples in contrast with the paired non-tumor samples (Figures 1A and 1B). Moreover, we also measured that circ-AKT1 was highly expressed in the serum of 58 CC patients, and low expressed in that of 40 healthy volunteers via quantitative real-time reverse transcription polymerase chain reaction (RT-PCR) (Figure 1C). Subsequently, ROC (receiver operating characteristic) curves were made, and the results indicated that circ-AKT1 may have diagnostic value for CC patients (Figure 1D). Therefore, these data confirmed that circ-AKT1 was upregulated in CC, indicating that circ-AKT1 might participate in CC.

circ-AKT1 Was a Bona Fide circRNA

Next, we tried to examine circular characteristics of circ-AKT1. We confirmed through circRNA sequencing that circ-AKT1 was backspliced from AKT1 gene, ranging from the 9th exon to the 13th exon of the host, which contains five exons (Figure 2A). Also, we found that, compared with AKT1, circAKT1 was resistant to the digestion by RNase R (Figure 2B, left), and that circ-AKT1 amplified by divergent primer was identified in complementary DNA (cDNA) rather than genomic DNA (gDNA) (Figure 2B, right). Besides, in Figure S2A, northern blot assay detected that the RNA expressions of GAPDH and AKT1 were decreased in RNase R treatment, whereas that of circ-AKT1 was not affected. Additionally, overexpression efficiency of circ-AKT1 was detected with or without Alu treatment (Figure S2B). Then we confirmed the elevated expression of circ-AKT1 in four CC cell lines (Figure 2C). Additionally, we examined the cellular localization of circ-AKT1. Subcellular fractionation analysis and fluorescence *in situ* hybridization (FISH) staining validated the concentrating distribution of circ-AKT1 in CC cell cytoplasm (Figures 2D and 2E). In general, these findings confirmed the circular structure and post-transcriptional regulation possibility of circ-AKT1 in CC.

circ-AKT1 Promoted Cell Proliferation and Invasion in CC

Then we explored the functional role of circ-AKT1 in CC through gain- and loss-of-function experiments. Because previously we found that circ-AKT1 presented the lowest expression in SiHa cells and highest in CaSki cells among four CC cells, we overexpressed circ-AKT1 in SiHa cells and knocked down circ-AKT1 in CaSki cells. Quantitative real-time RT-PCR results validated the upregulation of circ-AKT1 by pcDNA3.1/circ-AKT1 and the knockdown of circ-AKT1 by three specific siRNAs. In addition, siRNA#1 and siRNA#2 exhibited better knockdown efficiency (Figure 3A). Therefore, we used siRNA#1 and siRNA#2 for loss-of-function experiments. Results of Cell Counting Kit-8 (CCK-8) and colony formation assays

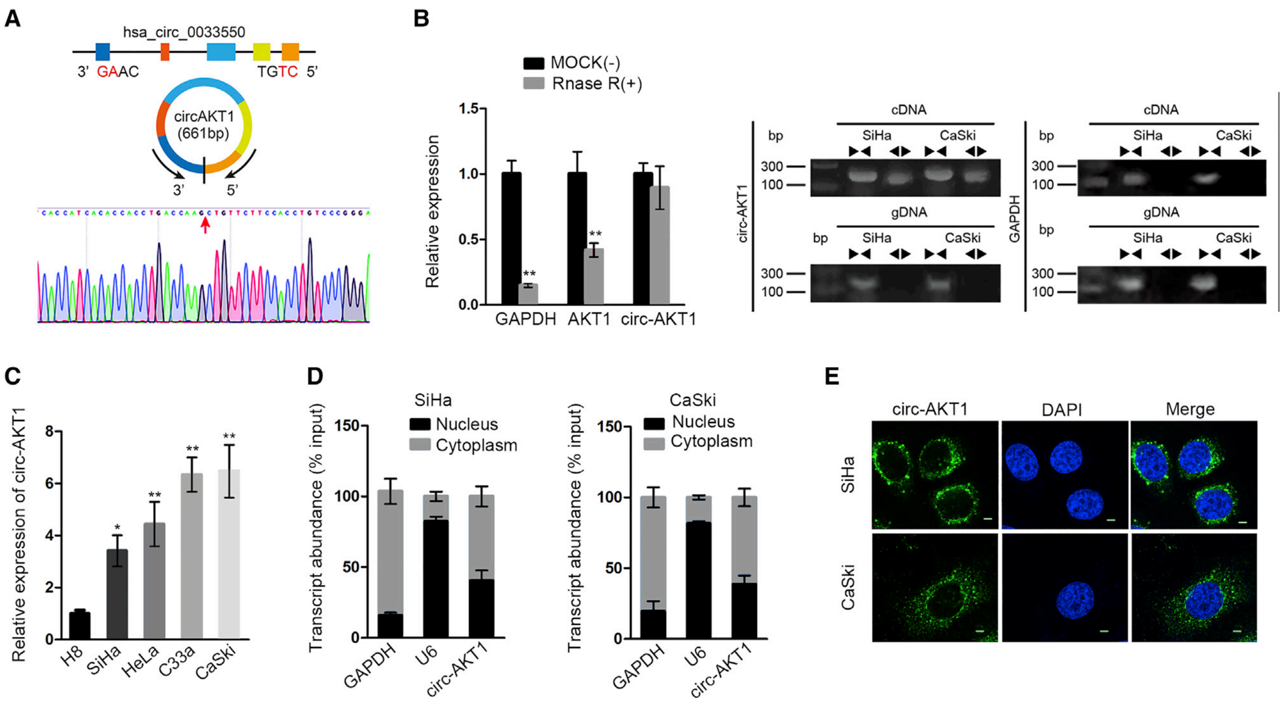

Figure 2. circ-AKT1 Was a Bona Fide circRNA

(A) circRNA sequencing analysis displayed the transcription process of circ-AKT1. (B) Quantitative real-time RT-PCR measured relative expression of circ-AKT1 and AKT1 in the RNase R-treated group. GAPDH was the negative control (left); PCR results measured the amplification primer of circ-AKT1 in cDNA and gDNA (right). (C) Relative expression of circ-AKT1 in CC cell lines was tested by quantitative real-time RT-PCR. (D) Subcellular fractionation assay detected transcript abundance of circ-AKT1 in cytoplasm and nucleus of SiHa and CaSki cells. (E) FISH staining confirmed the expression of circ-AKT1 in cytoplasm (scale bars, 10 μm). *$p < 0.05$, **$p < 0.01$.

displayed that CC cell proliferation was facilitated by circ-AKT1 overexpression but retarded by circ-AKT1 knockdown (Figures 3B and 3C). Also, 5-ethynyl-2′-deoxyuridine (EdU) assay demonstrated that the proliferative cells were increased by circ-AKT1 overexpression and were decreased by circ-AKT1 knockdown (Figure 3D). Transwell invasion assay showed that overexpression of circ-AKT1 enhanced invasive ability of CC cells, and that knockdown of circ-AKT1 led to opposite results (Figure 3E). On the whole, these data suggested that circ-AKT1 promoted cell proliferation and invasion in CC.

AKT1 Was Upregulated in CC and Promoted Proliferation and Invasion

Additionally, we tested the effect of AKT1 on CC development. We confirmed the high expression of AKT1 in CC cell lines and tissues (Figures 4A and 4B). In CC samples, we verified the positive correlation between AKT1 and circ-AKT1 (Figure 4C). We then knocked down AKT1 in CaSki cells, which was confirmed by quantitative real-time RT-PCR results (Figure 4D). We chose si-AKT1#1 and si-AKT1#2 for subsequent assays because they present better knockdown efficiency. CCK-8 and EdU assays illustrated that silencing AKT1 attenuated proliferative capacity of CC cells (Figures 4E and 4F). Also, knockdown of AKT1 decreased the colony number in CaSki cells (Figure 4G). Transwell invasion assay revealed that CC cells with AKT1 depletion presented weaker invasive ability than control (Figure 4H). In summary, the results above suggested that AKT1, positively correlated with circ-AKT1, was upregulated in CC cells and promoted cell proliferation and invasion.

TGF-β-Induced circ-AKT1 Upregulated AKT1 and Promoted EMT in CC

It has been known that transforming growth factor beta (TGF-β) is an upstream inducer of AKT1;[34,35] in addition, quantitative real-time RT-PCR detected that the expression of TGF-β was more upregulated in CC tissues and cells compared with that in corresponding control groups (Figures S3A and 3B). Therefore, we probed whether TGF-β was also responsible for the upregulation of circ-AKT1 in CC. After treating SiHa cells with TGF-β at the dose of 0, 1, 2, and 5 ng/mL, we detected the expressions of circ-AKT1 and AKT1. The results showed that the expressions of both AKT1 and circ-AKT1 were dose dependently increased by TGF-β (Figure 5A). Previous reports have illustrated that AKT1 regulated EMT progress in human cancers[36,37] and facilitated EMT progress, which enhanced migratory and invasive capacities of cancer cells.[38] Therefore, we examined the effect of TGF-β on the expressions of EMT markers. Levels of AKT1 and mesenchymal marker N-cadherin were increased, whereas that of epithelial marker E-cadherin was decreased by TGF-β in a dose-dependent manner (Figure 5B).

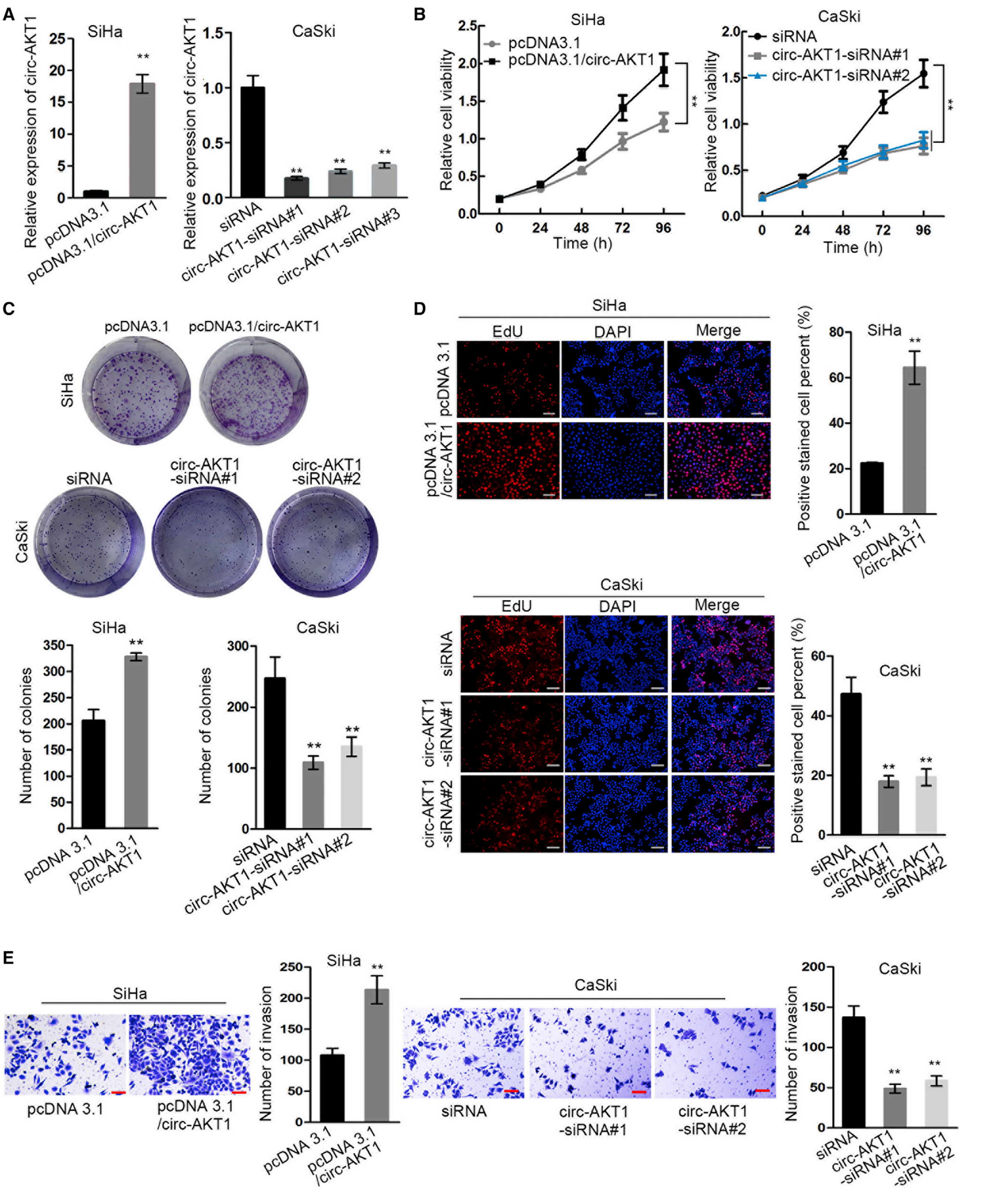

(legend on next page)

Furthermore, we detected the effect of circ-AKT1 on AKT1 expression. Results of quantitative real-time RT-PCR showed the increased AKT1 level upon circ-AKT1 overexpression and the decreased AKT1 level upon circ-AKT1 knockdown (Figure 5C). Later, immunofluorescence (IF) staining exhibited that overexpression of circ-AKT1 increased N-cadherin and decreased E-cadherin expression, whereas silencing circ-AKT1 exhibited the opposite effects (Figure 5D). The same results were observed through western blot analysis as well (Figure 5E). Generally speaking, these results indicated that TGF-β-induced circ-AKT1 upregulated AKT1 and promoted EMT in CC.

circ-AKT1 Sequestered miR-942-5p to Regulate AKT1 in CC

Plentiful studies have demonstrated that circRNAs are able to function through competing endogenous RNA (ceRNA) mechanism in cancers, whereby they could sequester miRNAs to induce downstream gene expression.[12] Therefore, we planned to confirm whether circ-AKT1 could regulate AKT1 expression through targeting miRNAs. We searched for the miRNAs potentially interacting with both circ-AKT1 and AKT1. We found 107 putative miRNAs targeting AKT1 from PITA, and 20 miRNAs targeted by circ-AKT1 from CircInteractome. The intersection of the findings from the two databases contained two miRNAs (miR-942-5p and miR-338-3p) (Figure 6A). To detect the involvement of these two miRNAs in CC, we examined their expressions in cell lines. As a result, only miR-942-5p was downregulated in CC cell lines compared with the normal cell line (Figure 6B), indicating that miR-942-5p was associated with CC. Therefore, we focused on the investigation of miR-942-5p in the following experiments. Quantitative real-time RT-PCR validated that overexpression of circ-AKT1 reduced the level of miR-942-5p, whereas knockdown of circ-AKT1 resulted in the opposite conclusion (Figure 6C). Additionally, miR-942-5p was downregulated in CC samples and negatively correlated with circ-AKT1 and AKT1 expressions (Figure 6D).

Subsequently, we examined the interaction of miR-942-5p with circ-AKT1 and AKT1. The binding sequences on circ-AKT1 and AKT1 for miR-942-5p were presented in Figure 6E. RNA pull-down assay revealed that miR-942-5p could be pulled down by the biotin-circ-AKT1 probe instead of the no biotin probe (Figure 6F). RNA immunoprecipitation (RIP) assay validated that circ-AKT1, miR-942-5p, and AKT1 could be co-immunoprecipitated by anti-Ago2 (Figure 6G). Luciferase reporter assay demonstrated that overexpressing miR-942-5p decreased the luciferase activity of circ-AKT WT (wild-type) and AKT1 WT. Meanwhile, that of circ-AKT MUT (mutant) and AKT1 MUT was not affected (Figure 6H). Therefore, these results suggested that circ-AKT1 sequestered miR-942-5p to regulate AKT1 in CC.

circ-AKT1 Regulated CC Progression through the miR-942-5p/AKT1 Axis

In order to explore whether circ-AKT1 regulated CC progression through the miR-942-5p/AKT1 axis, we performed rescue assays. AKT1 expression decreased by circ-AKT1 knockdown was restored by miR-942-5p inhibitor, but such restoration could be impaired by silencing AKT1 in CaSki cells (Figure 7A). Western blot analyses showed that the increased expression of E-cadherin or decreased expressions of N-cadherin and AKT1 caused by circ-AKT1 silence could be reversed by miR-942-5p inhibitor, but such reversing effect could be counteracted by AKT1 knockdown (Figure 7B). Moreover, the CC cell proliferation and invasion attenuated by circ-AKT1 knockdown were restored by miR-942-5p inhibition, and similarly, such restoration role could be abrogated by AKT1 depletion (Figures 7C–7F). Altogether, it was indicated that circ-AKT1 regulated CC progression through the miR-942-5p/AKT1 axis.

circ-AKT1 Facilitated CC Progression *In Vivo*

Finally, we examined the effect of circ-AKT1 on the tumor growth of CC in animal models. circ-AKT1 was overexpressed in SiHa cells, and cells were injected into nude mice to generate xenografts. The tumor volume was detected over time. It turned out that injecting SiHa cells with circ-AKT1 overexpression into mice caused the generation of larger tumors compared with control (Figure 8A). Also, the tumor growth in mice was facilitated by circ-AKT1 overexpression (Figure 8B). Then we resected the tumors and validated that overexpression of circ-AKT1 could increase the volume and weight of xenografts in mice (Figure 8C). Additionally, we confirmed that expressions of circ-AKT1 and AKT1 were increased, whereas expression of miR-942-5p was reduced by circ-AKT1 overexpression *in vivo* (Figure 8D). Moreover, the positive correlation between circ-AKT1 and AKT1, and the negative correlation of miR-942-5p with circ-AKT1 and AKT1 were confirmed in the tumors from mice injected with SiHa cells/pcDNA3.1/circ-AKT1 (Figure 8E). Results of western blot assay demonstrated that the levels of AKT1 and N-cadherin were elevated, whereas the level of E-cadherin was decreased in mice injected with SiHa/pcDNA3.1/circ-AKT1 (Figure 8F). In conclusion, circ-AKT1 facilitated CC progression *in vivo*.

DISCUSSION

Emerging studies have shown that circRNAs are not merely splicing errors, as initially regarded,[7] but are key regulators for the progression of cancers,[10,11] including CC.[12] Therefore, identification of new circRNAs participating in CC progression could provide promising treatment targets for CC patients.

Herein, we performed circRNA microarray analysis and picked the top 50 upregulated circRNAs in CC samples. Among them, AKT1

Figure 3. circ-AKT1 Promoted Cell Proliferation and Invasion in CC

(A) Quantitative real-time RT-PCR detected relative expression of circ-AKT1 in pcDNA3.1/circ-AKT1-transfected SiHa cells and circ-AKT1-siRNA#1-, circ-AKT1-siRNA#2-, or circ-AKT1-siRNA#3-transfected CaSki cells. (B) CCK-8 detected SiHa and CaSki cell viability in differently transfected conditions. (C) Colony formation assay measured colony number of transfected SiHa and CaSki cells. (D) EdU assay detected positive stained cell percent when overexpressing or knocking down circ-AKT1 (scale bars, 100 μm). (E) Transwell invasion assay detected the invasive ability of SiHa and CaSki cells upon circ-AKT1 overexpression and knockdown (scale bars, 60 μm). $^{**}p < 0.01$.

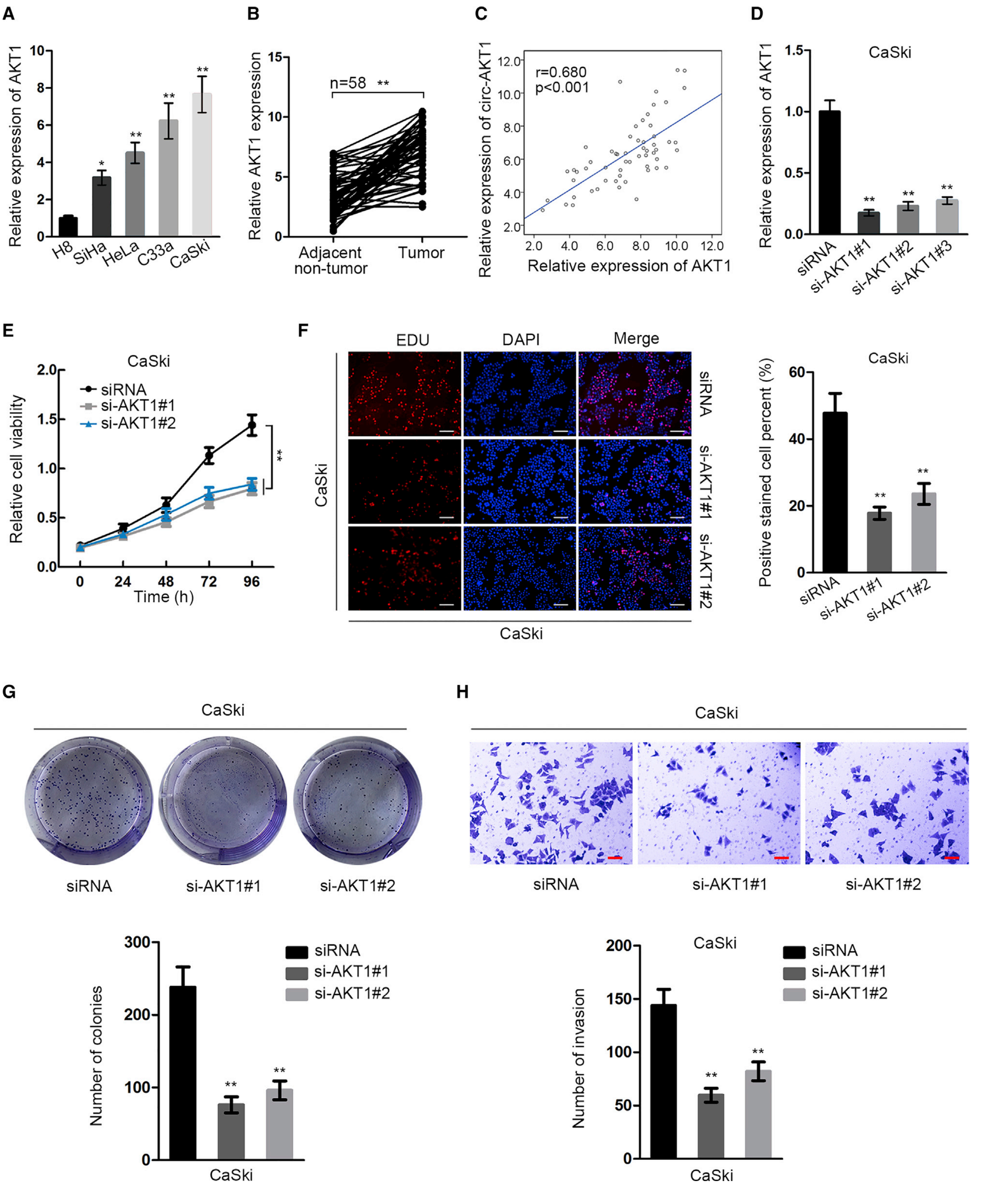

(legend on next page)

was the associated gene of circ-AKT1. Moreover, we validated that circ-AKT1 expression was elevated in CC tissues and cells, indicating the involvement of circ-AKT1 in CC. Besides, circular characteristics and cytoplasmic localization of circ-AKT1 were verified, suggesting that circ-AKT1 was a bona fide circRNA. Gain- and loss-of-function experiments suggested that circ-AKT1 promoted proliferation and invasion of CC cells.

Based on the findings that AKT1 was a host gene for circ-AKT1, we speculated the regulation of circ-AKT1 on AKT1. AKT1 is an important factor involved in the PI3K/AKT pathway,[17,18] and it could regulate multiple cellular processes, such as proliferation, invasion, and EMT.[19–21,36] Concordant with previous findings, AKT1 exerted oncogenic functions in CC.[22,23] Our study also validated that AKT1 was upregulated in CC tissues and cell lines, and that silencing AKT1 inhibited cell proliferation and invasion. Additionally, it has been revealed that TGF-β is an upstream activator of AKT1,[34,35] so we hypothesized that TGF-β was responsible for the induction of circ-AKT1 as well. Furthermore, TGF-β could induce the expression of circ-AKT1 and AKT1 in a dose-dependent manner, and that circ-AKT1 positively regulated AKT1 expression, indicating that TGF-β could stimulate AKT1 expression through circ-AKT1. Additionally, previous reports have illustrated that AKT1 could modulate EMT progress in human cancers.[36,37] In this research, we confirmed that circ-AKT1 promoted the EMT process in CC. The above findings suggested the effects of the TGF-β/circ-AKT1/AKT1 axis on CC progression.

With regard to the molecular regulatory mechanism, circRNAs can regulate target gene expression through performing as sequesters of miRNAs in cancers,[24,25] including in CC.[12] In the present study, we identified two shared miRNAs for circ-AKT1 and AKT1 through bioinformatics tools, but found that only miR-942-5p was downregulated in CC cell lines and tissues. Moreover, a former study revealed that miR-942-5p was a tumor suppressor in KSHV-associated malignancies.[31] In accordance, our findings first suggested that miR-942-5p was a tumor suppressor gene in CC. Further, the negative correlation of miR-942-5p with circ-AKT1 and AKT1 in CC samples was demonstrated. In addition, we verified the interaction of miR-942-5p with circ-AKT1 and AKT1 in CC. circ-AKT1 could modulate AKT1 expression through sponging miR-942-5p. Meanwhile, rescue assays elucidated that circ-AKT1 regulated CC progression through the miR-942-5p/AKT1 axis. Finally, *in vivo* assays validated that circ-AKT1 promoted tumor growth and regulated miR-942-5p/AKT1 expression in CC.

In conclusion, our study was the first to discover the oncogenic function of circ-AKT1 in CC, which demonstrated the mechanism that circ-AKT1 was induced by TGF-β and could sequester miR-942-5p to upregulate AKT1 expression in CC (Figure 8G). Our findings indicated circ-AKT1 as a novel promising therapeutic target for CC treatment.

MATERIALS AND METHODS

Tissue and Serum Samples

CC tissues (n = 58) and the paired adjacent non-cancerous tissues (n = 58) were provided by the First Affiliated Hospital of Wenzhou Medical University. In addition, serum samples from CC patients (n = 58) and that from corresponding healthy volunteers (n = 40) were collected. All CC patients underwent no chemotherapy or radiotherapy prior to biopsy or surgical treatment. The collected tissue samples were maintained instantly at −80°C following resection. The permission for this study was obtained from the Ethics Committee of the First Affiliated Hospital of Wenzhou Medical University. Informed consents were signed by all patients enrolled.

circRNA Microarray Analysis

Three pairs of CC tissues and the matched non-cancerous tissues were picked for microarray analysis. Extraction of RNA and hybridization of microarray were implemented obeying the Arraystar's standard protocols. To be brief, RNase R (Epicenter, Madison, WI, USA) was used for RNA digestion for the removal of linear RNA and enrichment of circRNA. Thereafter, RNAs underwent amplification to generate cDNA and went through labeling by the Arraystar Human Circular RNA Array (AS-S-CR-H-V2.0) (Arraystar, Rockville, MD, USA). Eventually, the labeled RNAs were subjected to hybridization with the application of Arraystar mouse circRNA Array (v.1.0; Arraystar), and Agilent Scanner G2505C was used for the scanning.

Cell Lines and Cell Culture

CC cell lines (HeLa, CaSki, SiHa, and C33A) and 293T cells were provided by the American Type Culture Collection (ATCC, Rockville, MD, USA), and cervical epithelial immortalized cells (H8) were provided by the Cell Resource Database of Chinese Academy of Sciences (Shanghai, China). HeLa, SiHa, C33A, and 293T cells were cultured in Dulbecco's modified Eagle's medium (DMEM; GIBCO-BRL; Rockville, MD, USA). CaSki and H8 cells were cultured in Roswell Park Memorial Institute (RPMI)-1640 medium (HyClone, Logan, UT, USA). All mediums were kept in an air at 37°C containing 5% CO_2. 10% fetal bovine serum (FBS; GIBCO BRL, Grand Island, NY, USA) was used to supplement the media.

Cell Transfection

circ-AKT1 was silenced by specific small interfering RNAs (circ-AKT1-siRNA#1, circ-AKT1-siRNA#2, and circ-AKT1-siRNA#3)

Figure 4. AKT1 Was Upregulated in CC Cells and Promoted Proliferation and Invasion

(A and B) Quantitative real-time RT-PCR detected relative expression of AKT1 in CC cell lines and tissues. (C) Spearman's correlation analysis was used to confirm the correlation between circ-AKT1 and AKT1 expressions in CC samples. (D) Quantitative real-time RT-PCR measured relative expression of AKT1 in si-AKT1#1-, si-AKT1#2-, and si-AKT1#3-transfected CaSki cells. (E) CCK-8 tested cell viability of CaSki cells. (F) EdU assay detected positive stained cell percent (scale bars, 100 μm). (G) Colony formation assay measured colony number in differently transfected groups. (H) Transwell invasion tested the number of invasion SiHa and CaSki cells in response to the transfection of si-AKT1#1 or si-AKT1#2 compared with control (scale bars, 60 μm). $^{*}p < 0.05$, $^{**}p < 0.01$.

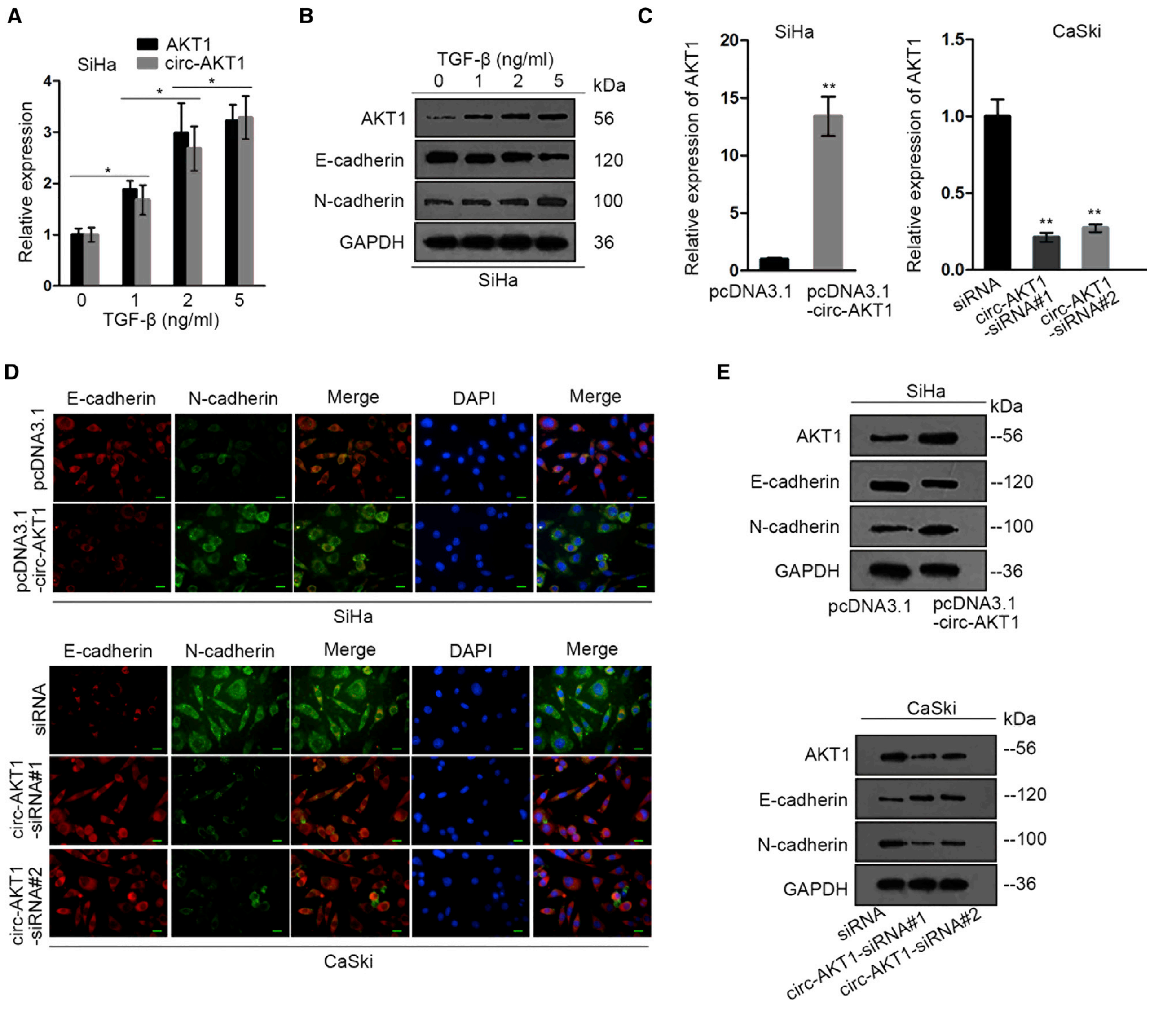

Figure 5. TGF-β-Induced circ-AKT1 Upregulated AKT1 and Promoted EMT in CC

(A) Quantitative real-time RT-PCR detected the expressions of circ-AKT1 and AKT1 in SiHa cells treated with TGF-β at the dose of 0, 1, 2, and 5 ng/mL. (B) Western blot assay displayed the expressions of AKT1, E-cadherin, and N-cadherin in SiHa cells treated with TGF-β at the dose of 0, 1, 2, and 5 ng/mL. (C) Quantitative real-time RT-PCR measured the expression of AKT1 in SiHa and CaSki cells upon circ-AKT1 overexpression and knockdown. (D) IF (immunofluorescence) staining detected the fluorescence intensities of E-cadherin and N-cadherin in SiHa and CaSki cells upon circ-AKT1 overexpression and knockdown (scale bars, 40 μm). (E) Western blot assay tested the expressions of AKT1, E-cadherin, and N-cadherin in SiHa and CaSki cells upon circ-AKT1 overexpression and knockdown. *p < 0.05, **p < 0.01.

and was overexpressed by pcDNA3.1 (Invitrogen, Carlsbad, CA, USA) integrated with full-length sequences of circ-AKT1. AKT1 was silenced by siRNAs targeting AKT1 (si-AKT1#1, si-AKT1#2, and si-AKT1#3). Scramble siRNAs (si-NC) and empty pcDNA3.1 vectors were negative controls (NCs). The overexpression and inhibition of miR-942-5p were realized by miR-942-5p mimics and miR-942-5p inhibitor, with NC mimics and NC inhibitor as controls (provided by Thermo Scientific, Waltham, MA, USA). The transfection of these plasmids into SiHa and CaSki cells was carried out with Lipofectamine 3000 Reagent (Thermo Fisher Scientific, CA, USA) as required, and sequences of the above-mentioned plasmids were provided in Table S1.

Quantitative Real-Time RT-PCR

Isolation of RNAs from CC samples and cells was accomplished by using TRIzol reagent (Invitrogen, Carlsbad, CA, USA). Generation of cDNA from isolated RNAs was carried out using a reverse transcriptase kit (Takara, Tokyo, Japan) or Thermo Fisher's K1622 kit (Thermo Fisher Scientific, USA). SYBR-Green PCR Master Mix kit (Takara, Dalian, China) was applied for quantitative real-time

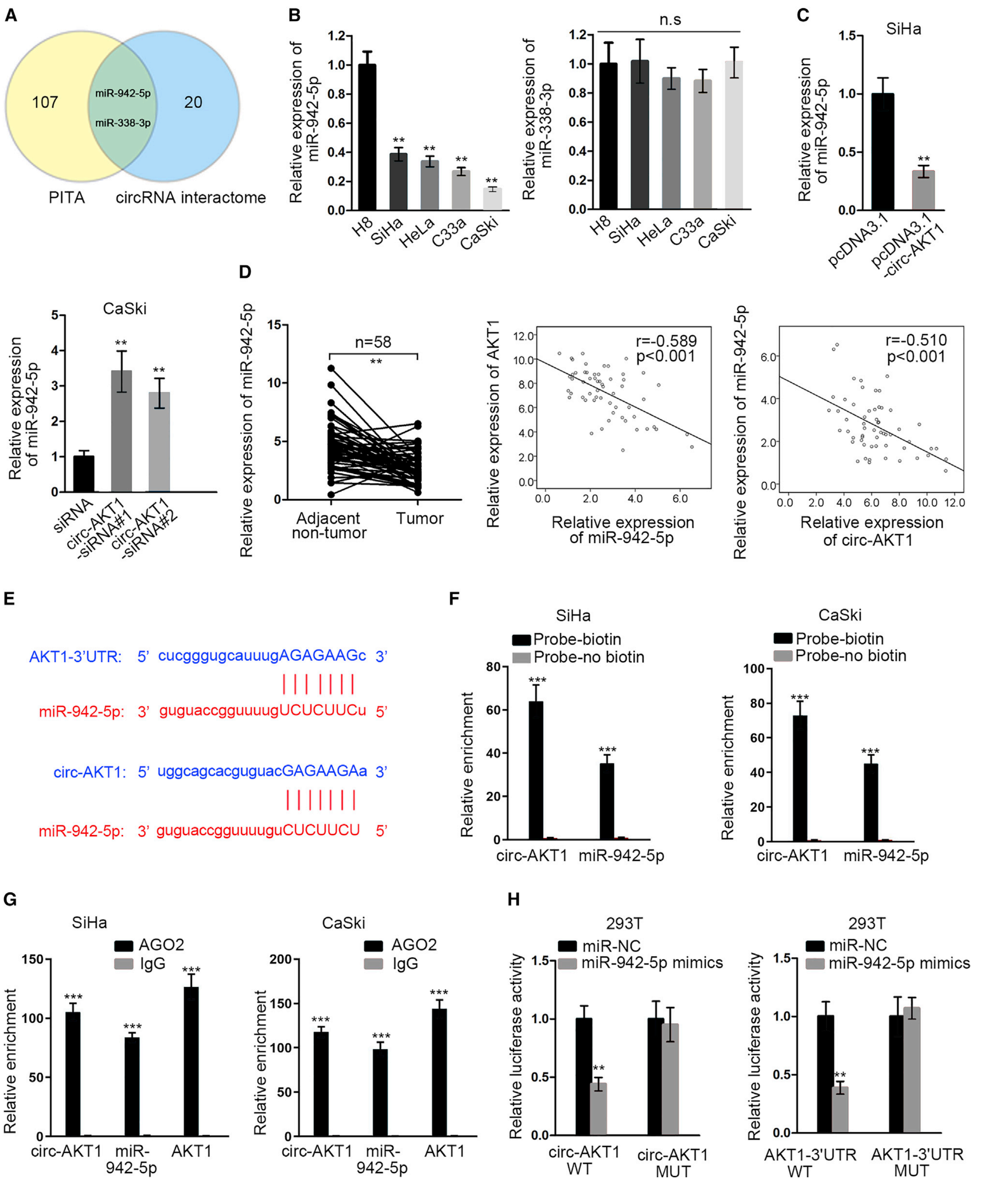

(legend on next page)

RT-PCR. GAPDH and U6 were the endogenous controls. Evaluation of gene expression was based on the $2^{-\Delta\Delta Ct}$ method. The primers for circ-AKT1, miR-942-5p, and AKT1 were obtained from Sangon Biotech (Shanghai, China). Related primer sequences were provided in Table S1.

Circular Structure Confirmation

The circular characteristic of circ-AKT1 was examined by Sanger sequencing and PCR with the use of divergent primers and RNase R treatment. The PCR products were amplified by the divergent primers of circ-AKT1 and then integrated into the T vector, and the genomic region for circAKT1 with its flanking introns containing two inverted repeats (AluJr and AluSz) was amplified and inserted into pGEM-T vector. The Sanger sequencing was conducted by Tsingke Biotech. Then results were cross-checked by the use of the backspliced region of circ-AKT1 obtained from circBASE. Additionally, because circRNAs were normally generated from pre-mRNAs, the convergent and divergent primers were applied to amplify the linear and circular transcripts of AKT1 in both gDNA and cDNA from CC cells. Theoretically, circular transcripts of AKT1 could be amplified only by the divergent primers in cDNA rather than in gDNA. The NC was GAPDH. For the RNase R treatment, RNAs extracted from CC cells were incubated for 45 min at 37°C with the addition of 10 U RNase R (20 U/μL; Epicenter, Madison, WI, USA) in 10 μL volume, followed by the incubation at 70°C for 10 min for the deactivation of RNase R. Thereafter, the treated RNAs were analyzed by quantitative real-time RT-PCR.

Northern Blot Assay

Northern blotting was carried out by utilizing the DIG Northern Starter Kit (Roche Diagnostics). The probed membrane was washed two times by using 2× SSC (300 mM NaCl, 30 mM sodium citrate [pH 7.0]) supplemented with 0.1% sodium dodecyl sulfate (SDS) for 6 min at room temperature and another two times with 0.1× SSC containing 0.1% SDS for 16 min at 69°C. Horseradish peroxidase (HRP)-conjugated anti-digoxigenin was applied to measure the hybridized probe by chemiluminescent imaging with a lumino-image analyzer (LAS-4000; GE Health Care, Hino, Tokyo, Japan), and GAPDH was the NC.

CCK-8 Assay

CC cell proliferation was evaluated by CCK-8 (Dojindo, Japan). In brief, 1×10^3 CC cells (SiHa and CaSki) were plated into each well of 96-well plates, and CCK-8 solution (10 μL) was added to each well after cells were incubated for 0, 24, 48, 72, and 96 h, followed by the incubation of 2 h. The absorbance was evaluated at 450 nm using a spectrophotometer.

EdU Assay

EdU assay was implemented with a Cell-Light EdU DNA Cell Proliferation Kit (RiboBio, Guangzhou, China). After being incubated with EdU for 3 h, the cells were fixed and went through nuclei staining with 4,6-diamidino-2-phenylindole (DAPI; Sigma-Aldrich, St. Louis, MO, USA). Images were captured with the inverted fluorescence microscope (Carl Zeiss, Jena, Germany). The ratio of EdU-positive cells was evaluated.

Colony Formation Assay

Cells were seeded into the six-well plates (5×10^2 cells/well) and underwent incubation for 14 days. Thereafter, cells were fixed in methanol and subjected to crystal violet staining. The colonies consisting of over 50 cells were counted under a microscope.

Transwell Invasion Assay

Cell invasion was examined by utilizing Corning Polycarbonate Membrane Insertin transwell chamber (Corning Costar, Cambridge, MA, USA). After 24 h of transfection, CC cells were placed in the upper compartment pre-coated with Matrigel (Sigma-Aldrich, St. Louis, MO, USA) in non-serum medium. 20% FBS was used to supplement the medium in the lower compartment as a chemoattractant. After incubation for 2 days, the cells that had not invaded were removed by cotton swabs. Cells invading to the bottom of the membrane were fixed and underwent staining using 0.1% crystal violet. The pictures were captured utilizing the microscope. The number of cells was evaluated by ImageJ software.

FISH Assay

After the culturing on coverslips, fixation, and permeabilization, the coverslips went through hybridization overnight utilizing hybridization buffer (Geneseed Biotech, Guangzhou, China) with the addition of digoxin as well as biotin-labeled single-stranded DNA probes at 37°C. Digoxin-labeled probes for circ-AKT1 were obtained from GenePharma (Shanghai, China). Detection of signals was conducted by using Cy3-conjugated anti-digoxin, as well as fluorescein isothiocyanate (FITC)-conjugated anti-biotin antibodies (Jackson ImmunoResearch, West Grove, PA, USA). Then cell nuclei underwent counterstaining by utilizing DAPI. Images were captured by utilizing Zeiss LSM 700 confocal microscope (Carl Zeiss, Oberkochen, Germany).

Figure 6. circ-AKT1 Sequestered miR-942-5p to Regulate AKT1 in CC

(A) The Venn diagram showed that miR-942-5p and miR-338-3p were in the intersection of prediction results of PITA and CircInteractome databases. (B) Quantitative real-time RT-PCR detected relative expression of miR-942-5p and miR-338-3p in CC cell lines. (C) Quantitative real-time RT-PCR was used to detect the expression of miR-942-5p upon circ-AKT1 overexpression and knockdown in SiHa and CaSki cells. (D) Quantitative real-time RT-PCR measured the expression of miR-942-5p in CC samples (left). Spearman's correlation curve showed the correlation of miR-942-5p with circ-AKT1 and AKT1 (right). (E) The binding sites between AKT1 and miR-942-5p, and the binding sites between circ-AKT1 and miR-942-5p were predicted by starBase. (F) Pull-down assay detected the input recovery of circ-AKT1 and miR-942-5p in SiHa and CaSki cells. (G) RIP assays measured the input recovery of circ-AKT1, miR-942-5p, and AKT1 in SiHa and CaSki cells. (H) Luciferase reporter assay was used to examine the luciferase activity of circ-AKT1-WT and circ-AKT1-MUT in transfected 293T cells. **$p < 0.01$, ***$p < 0.001$.

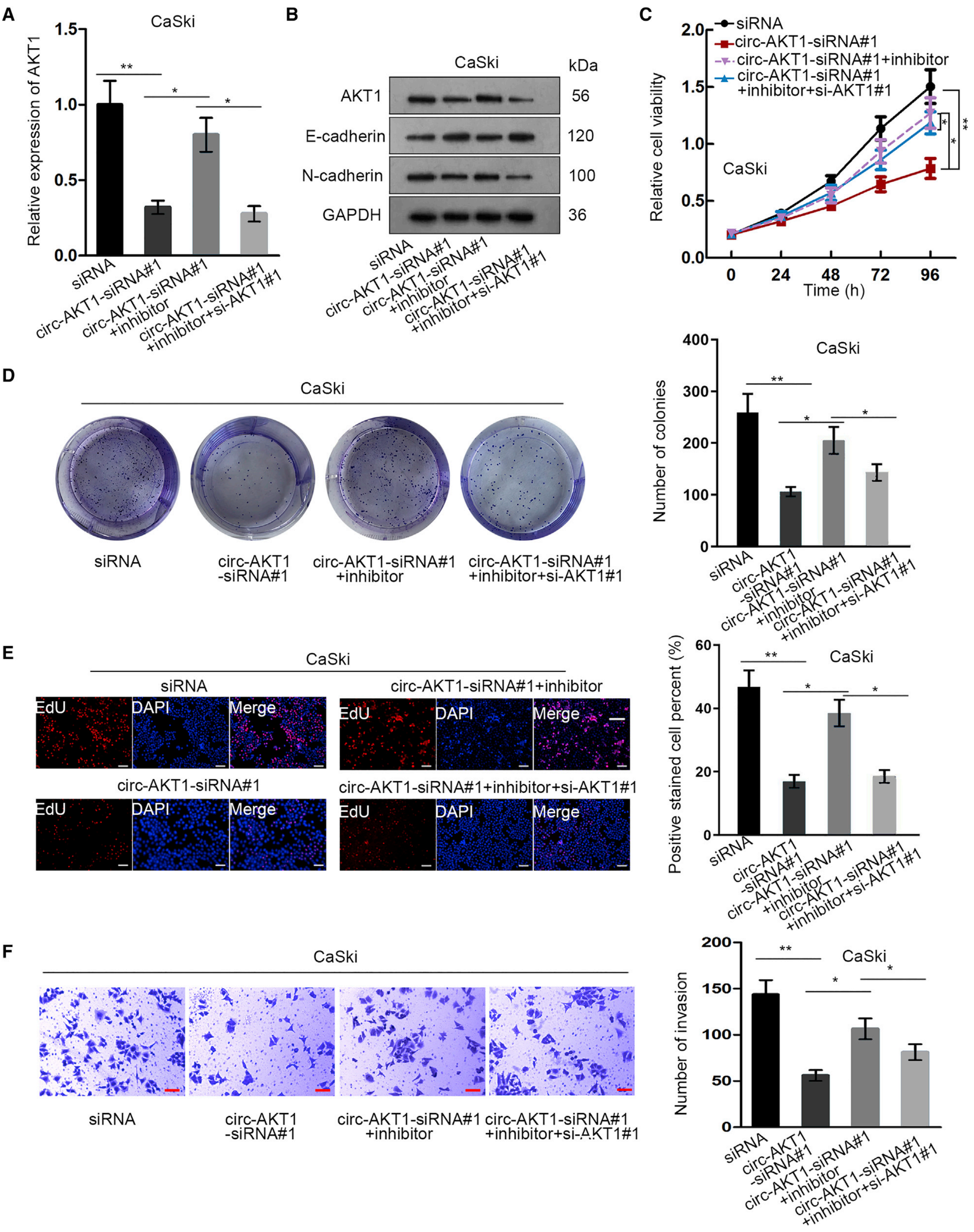

(legend on next page)

IF Staining Assay

After the fixation in 4% formaldehyde solution and the wash in PBS, CC cells were lysed utilizing 0.5% Triton-X. Then cells were subjected to incubation utilizing 5% BSA for 5 min to prevent non-specific background staining. Thereafter, cells underwent incubation with the primary antibodies against E-cadherin and N-cadherin for 2 h at 37°C, followed by incubation for 1 h with the secondary antibody. Later, cells were subjected to the treatment of DAPI for 10 min. The fluorescence microscope was used to observe the cells.

RNA Pull-Down Assay

In brief, transcripts of circ-AKT1 were transcribed utilizing T7 RNA polymerase (Ambion Life, Taoyuan, Taiwan). The purified RNAs were biotin labeled by Biotin RNA Labeling Mix (Ambion Life). The cell lysates[39] were incubated with the biotin-labeled or non-biotin-labeled probes of circ-AKT1. Streptavidin magnetic beads were then added to prepare the probe-magnetic bead complexes. After washing the bead complexes, the eluted RNAs were examined by quantitative real-time RT-PCR.

Subcellular Fractionation

Separation of cytoplasmic and nuclear RNA in SiHa and CaSki cells was carried out using Cytoplasmic and Nuclear RNA Purification Kit (Norgenbiotek Corporation, Thorold, ON, Canada). The relative expression level of circ-AKT1 was subjected to quantitative real-time RT-PCR. U6 (nucleus control) and GAPDH (cytoplasm control) were employed for normalization.

RIP

The RIP assay was implemented with the application of Magna RIP RNA Binding Protein Immunoprecipitation Kit (Bersinbio, Guangzhou, China). After being lysed in the complete RIP lysis buffer, cell lysates were obtained and separated into two equal parts for the overnight incubation with antibody against anti-Argonaute2 (Ago2) (Millipore, Billerica, MA, USA) or IgG (Millipore) with rotation at 4°C. Then the cell lysates were incubated for 1 h with the addition of magnetic beads. Thereafter, Proteinase K was added and samples underwent 1-h incubation. The expressions of purified RNAs were determined by quantitative real-time RT-PCR analysis.

Dual-Luciferase Reporter Assay

The 3′ UTR regions of AKT1 and circ-AKT1 containing binding sequences for miR-942-5p were generated into the pmirGLO luciferase vectors (Promega, Madison WI, USA) to generate circ-AKT1-WT and AKT1-WT reporters, and the circ-AKT1-MUT and AKT1-MUT reporters (with the MUT type of sequences) were generated as well. The circ-AKT1-WT, circ-AKT1-MUT, AKT1-WT, or AKT1-MUT was respectively transfected with miR-942-5p mimics or NC mimics into 293T cells using Lipofectamine 2000 (Invitrogen). Following transfection for 2 days, the luciferase activities were determined by the application of dual-luciferase reporter assay kit (Promega, Madison WI, USA), and relative luciferase activities were normalized to the Renilla luciferase activities.

Western Blot

Extracts of total proteins were obtained from cells with the use of radioimmunoprecipitation assay (RIPA) lysis buffer (Beyotime, Shanghai, China) with the supplementation of 1% PMSF (Roche, Basel, Switzerland). After the separation of equal quantity of protein samples by 10% SDS polyacrylamide gel electrophoresis (SDS-PAGE) gels, the proteins were shifted to polyvinylidene fluoride (PVDF) membranes, which were later sealed with skim milk. Blots were subjected to 12-h incubation with primary antibodies and then 1-h incubation with secondary antibody. Electrochemiluminescence (ECL) kit (Tanon) was applied for the visualization of protein signals. GAPDH was the internal control. Primary antibodies were anti-E-cadherin, anti-N-cadherin, anti-AKT1, anti-PCNA, and anti-Ki67 from Abcam (Cambridge, UK).

Xenograft Assay

The male BALB/c nude mice that were 4 weeks old were kept under non-pathogen conditions. The animal study procedures had gained the approval of the Animal Care Committee of The First Affiliated Hospital of Wenzhou Medical University. SiHa cells transfected with pcDNA3.1 or pcDNA3.1/circ-AKT1 were subcutaneously injected into either side of the flank area of the nude mice ($n = 5$ in each group) to generate the xenografts. The tumor growth was determined 7 days at a time. Tumor volume was evaluated based on the following equation: length $\times$ width^2 $\times$ 0.5. Twenty-eight days after injection, mice underwent euthanasia, and tumors were dissected for further assays.

Bioinformatics Tools

A total of 107 putative miRNAs targeting AKT1 were obtained from PITA (https://genie.weizmann.ac.il/pubs/mir07/mir07_data.html), and 20 miRNAs targeted by circ-AKT1 were provided by CircInteractome (http://circinteractome.nia.nih.gov/).

Statistical Analysis

Expression of quantitative data was based on mean ± standard deviation. Analysis of statistics was carried out on GraphPad Prism v.5.01 (GraphPad, La Jolla, CA, USA) software. Analysis of area under the ROC curve (AUC) was used to estimate the diagnostic significance of circ-AKT1. All experiments were performed at least three times.

Figure 7. circ-AKT1 Regulated CC Progression through the miR-942-5p/AKT1 Axis

CaSki cells were transfected with siRNA, circ-AKT1-siRNA#1, circ-AKT1-siRNA#1 + miR-942-5p inhibitor, or circ-AKT1-siRNA#1 + miR-942-5p inhibitor + si-AKT1#1 for subsequent assays. (A) Quantitative real-time RT-PCR detected AKT1 expression in each group. (B) Western blot assay measured the expressions of AKT1, E-cadherin, and N-cadherin in each group. (C) CCK-8 tested cell viability of CC cells in each group. (D) Colony formation assay measured colony number in differently transfected groups. (E) EdU assay detected positive stained cell percent in each group (scale bars, 100 μm). (F) Transwell invasion assay was used to detect the invasive ability of CC cells in each group (scale bars, 60 μm). *$p < 0.05$, **$p < 0.01$.

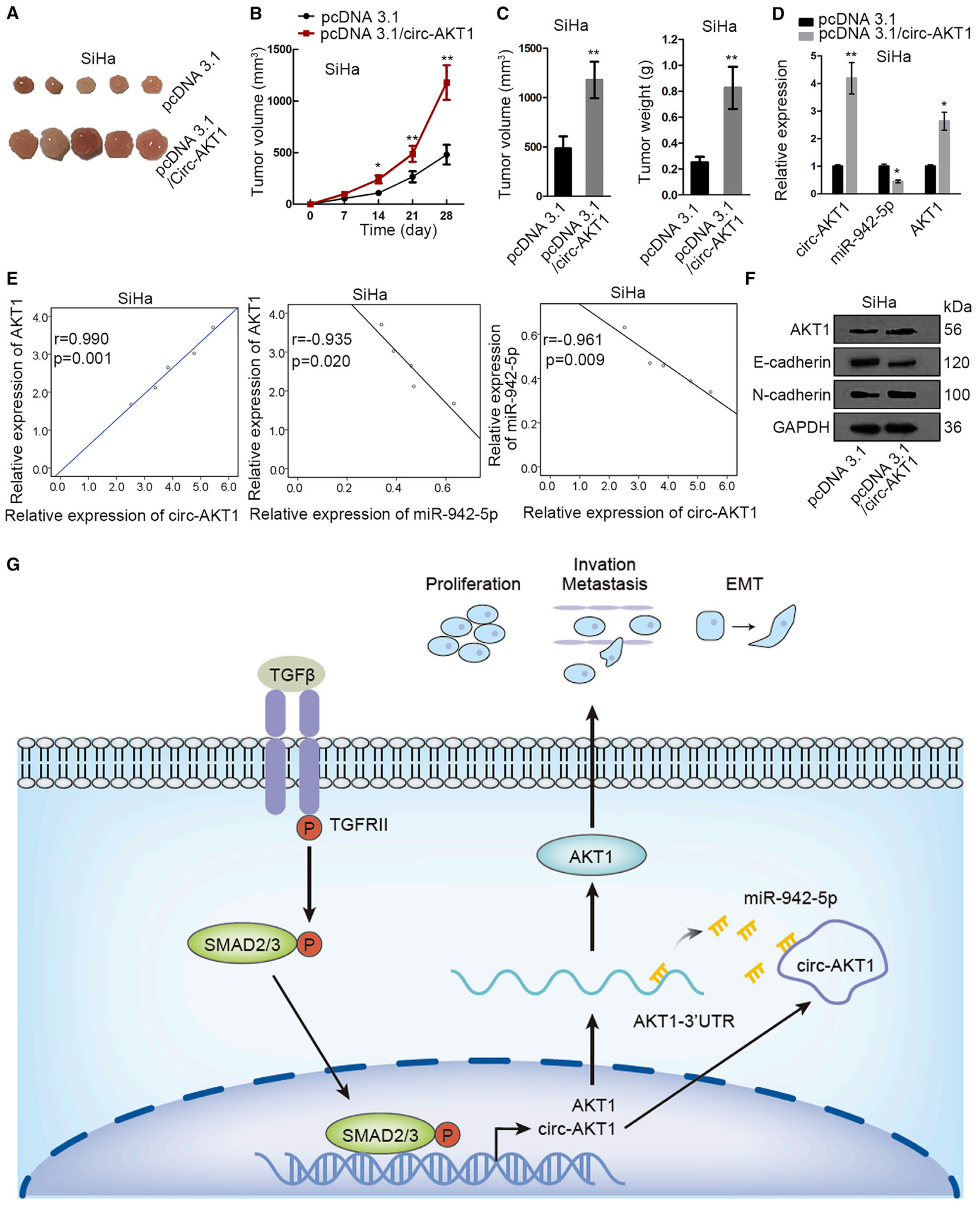

(legend on next page)

Student's t test (two-tailed) and one-way ANOVA were employed for comparing the differences between two groups or the differences among multiple groups. The statistical significance of differences was determined by $p < 0.05$.

SUPPLEMENTAL INFORMATION

Supplemental Information can be found online at https://doi.org/10.1016/j.omtn.2020.01.003.

AUTHOR CONTRIBUTIONS

R.O. and L.M. conceived and designed the study. H.T. and S.L. performed the experiments. H.Z. and L.Z. assisted with analyzing the data. R.O. drafted the manuscript, which was reviewed by Y.R. and Y.X.

CONFLICTS OF INTEREST

The authors declare no competing interests.

ACKNOWLEDGMENTS

We thank all involved in this study. This work was supported by grants from the National Natural Science Foundation of China (81571395, 81671408, 81771531, 81701634, and 81871129).

REFERENCES

1. Huang, Q.-T., Man, Q.-Q., Hu, J., Yang, Y.-L., Zhang, Y.-M., Wang, W., Zhong, M., and Yu, Y.H. (2017). Prognostic significance of neutrophil-to-lymphocyte ratio in cervical cancer: A systematic review and meta-analysis of observational studies. Oncotarget *8*, 16755–16764.
2. Bobdey, S., Sathwara, J., Jain, A., and Balasubramaniam, G. (2016). Burden of cervical cancer and role of screening in India. Indian J. Med. Paediatr. Oncol. *37*, 278–285.
3. Yang, W., Hong, L., Xu, X., Wang, Q., Huang, J., and Jiang, L. (2017). LncRNA GAS5 suppresses the tumorigenesis of cervical cancer by downregulating miR-196a and miR-205. Tumour Biol. *39*, 1010428317711315.
4. Ferlay, J., Soerjomataram, I., Dikshit, R., Eser, S., Mathers, C., Rebelo, M., Parkin, D.M., Forman, D., and Bray, F. (2015). Cancer incidence and mortality worldwide: sources, methods and major patterns in GLOBOCAN 2012. Int. J. Cancer *136*, E359–E386.
5. Panda, A.C., Grammatikakis, I., Munk, R., Gorospe, M., and Abdelmohsen, K. (2017). Emerging roles and context of circular RNAs. Wiley Interdiscip. Rev. RNA *8*, e1386.
6. Müller, S., and Appel, B. (2017). In vitro circularization of RNA. RNA Biol. *14*, 1018–1027.
7. Nigro, J.M., Cho, K.R., Fearon, E.R., Kern, S.E., Ruppert, J.M., Oliner, J.D., Kinzler, K.W., and Vogelstein, B. (1991). Scrambled exons. Cell *64*, 607–613.
8. Kulcheski, F.R., Christoff, A.P., and Margis, R. (2016). Circular RNAs are miRNA sponges and can be used as a new class of biomarker. J. Biotechnol. *238*, 42–51.
9. Meng, S., Zhou, H., Feng, Z., Xu, Z., Tang, Y., Li, P., and Wu, M. (2017). CircRNA: functions and properties of a novel potential biomarker for cancer. Mol. Cancer *16*, 94.
10. Kristensen, L.S., Hansen, T.B., Venø, M.T., and Kjems, J. (2018). Circular RNAs in cancer: opportunities and challenges in the field. Oncogene *37*, 555–565.
11. Chen, J., Li, Y., Zheng, Q., Bao, C., He, J., Chen, B., Lyu, D., Zheng, B., Xu, Y., Long, Z., et al. (2017). Circular RNA profile identifies circPVT1 as a proliferative factor and prognostic marker in gastric cancer. Cancer Lett. *388*, 208–219.
12. Ma, H.-B., Yao, Y.-N., Yu, J.-J., Chen, X.-X., and Li, H.-F. (2018). Extensive profiling of circular RNAs and the potential regulatory role of circRNA-000284 in cell proliferation and invasion of cervical cancer via sponging miR-506. Am. J. Transl. Res. *10*, 592–604.
13. Li, G., Yang, H., Han, K., Zhu, D., Lun, P., and Zhao, Y. (2018). A novel circular RNA, hsa_circ_0046701, promotes carcinogenesis by increasing the expression of miR-142-3p target ITGB8 in glioma. Biochem. Biophys. Res. Commun. *498*, 254–261.
14. Zhu, Q., Lu, G., Luo, Z., Gui, F., Wu, J., Zhang, D., and Ni, Y. (2018). CircRNA circ_0067934 promotes tumor growth and metastasis in hepatocellular carcinoma through regulation of miR-1324/FZD5/Wnt/β-catenin axis. Biochem. Biophys. Res. Commun. *497*, 626–632.
15. Ma, X., Yang, X., Bao, W., Li, S., Liang, S., Sun, Y., Zhao, Y., Wang, J., and Zhao, C. (2018). Circular RNA circMAN2B2 facilitates lung cancer cell proliferation and invasion via miR-1275/FOXK1 axis. Biochem. Biophys. Res. Commun. *498*, 1009–1015.
16. Conn, S.J., Pillman, K.A., Toubia, J., Conn, V.M., Salmanidis, M., Phillips, C.A., Roslan, S., Schreiber, A.W., Gregory, P.A., and Goodall, G.J. (2015). The RNA binding protein quaking regulates formation of circRNAs. Cell *160*, 1125–1134.
17. Emamian, E.S. (2012). AKT/GSK3 signaling pathway and schizophrenia. Front. Mol. Neurosci. *5*, 33.
18. Li, X., Kong, X., Wang, Y., and Yang, Q. (2013). BRCC2 inhibits breast cancer cell growth and metastasis in vitro and in vivo via downregulating AKT pathway. Cell Death Dis. *4*, e757.
19. Agarwal, E., Brattain, M.G., and Chowdhury, S. (2013). Cell survival and metastasis regulation by Akt signaling in colorectal cancer. Cell. Signal. *25*, 1711–1719.
20. Liu, H., Huang, X., Liu, X., Xiao, S., Zhang, Y., Xiang, T., Shen, X., Wang, G., and Sheng, B. (2014). miR-21 promotes human nucleus pulposus cell proliferation through PTEN/AKT signaling. Int. J. Mol. Sci. *15*, 4007–4018.
21. Saxena, N.K., Sharma, D., Ding, X., Lin, S., Marra, F., Merlin, D., and Anania, F.A. (2007). Concomitant activation of the JAK/STAT, PI3K/AKT, and ERK signaling is involved in leptin-mediated promotion of invasion and migration of hepatocellular carcinoma cells. Cancer Res. *67*, 2497–2507.
22. Cai, N., Wang, Y.-D., and Zheng, P.-S. (2013). The microRNA-302-367 cluster suppresses the proliferation of cervical carcinoma cells through the novel target AKT1. RNA *19*, 85–95.
23. Li, S., Ma, Y.-M., Zheng, P.-S., and Zhang, P. (2018). GDF15 promotes the proliferation of cervical cancer cells by phosphorylating AKT1 and Erk1/2 through the receptor ErbB2. J. Exp. Clin. Cancer Res. *37*, 80.
24. Hansen, T.B., Jensen, T.I., Clausen, B.H., Bramsen, J.B., Finsen, B., Damgaard, C.K., and Kjems, J. (2013). Natural RNA circles function as efficient microRNA sponges. Nature *495*, 384–388.

Figure 8. circ-AKT1 Facilitated CC Progression *In Vivo*

Nude mice were injected with SiHa cells transfected with pcDNA3.1 or pcDNA3.1/circ-AKT1. (A) Pictures of xenografts in mice injected with SiHa cells transfected with pcDNA3.1 or pcDNA3.1/circ-AKT1. (B) The growth curve of xenografts in mice injected with SiHa cells transfected with pcDNA3.1 or pcDNA3.1/circ-AKT1. (C) The final tumor volume and weight in mice injected with SiHa cells transfected with pcDNA3.1 or pcDNA3.1/circ-AKT1. (D) Quantitative real-time RT-PCR measured the expressions of circ-AKT1, AKT1, and miR-942-5p in mice injected with SiHa cells transfected with pcDNA3.1 or pcDNA3.1/circ-AKT1. (E) Spearman's correlation curve showed the correlation between circ-AKT1 and AKT1, and the correlation of miR-942-5p with circ-AKT1 and AKT1 in mice injected with SiHa cells transfected with pcDNA3.1/circ-AKT1. (F) Western blot detected the expressions of AKT1, E-cadherin, and N-cadherin in mice injected with SiHa cells transfected with pcDNA3.1 or pcDNA3.1/circ-AKT1. (G) circ-AKT1 was induced by TGF-β and could sequester miR-942-5p to upregulate AKT1 expression, so as to promote cell proliferation and invasion in CC. *$p < 0.05$, **$p < 0.01$.

25. Memczak, S., Jens, M., Elefsinioti, A., Torti, F., Krueger, J., Rybak, A., Maier, L., Mackowiak, S.D., Gregersen, L.H., Munschauer, M., et al. (2013). Circular RNAs are a large class of animal RNAs with regulatory potency. Nature *495*, 333–338.

26. Monroig, Pdel.C., Chen, L., Zhang, S., and Calin, G.A. (2015). Small molecule compounds targeting miRNAs for cancer therapy. Adv. Drug Deliv. Rev. *81*, 104–116.

27. Yang, Y., Song, K.L., Chang, H., and Chen, L. (2014). Decreased expression of microRNA-126 is associated with poor prognosis in patients with cervical cancer. Diagn. Pathol. *9*, 220.

28. Hao, Z., Yang, J., Wang, C., Li, Y., Zhang, Y., Dong, X., Zhou, L., Liu, J., Zhang, Y., and Qian, J. (2015). MicroRNA-7 inhibits metastasis and invasion through targeting focal adhesion kinase in cervical cancer. Int. J. Clin. Exp. Med. *8*, 480–487.

29. Smith, L., Baxter, E.W., Chambers, P.A., Green, C.A., Hanby, A.M., Hughes, T.A., Nash, C.E., Millican-Slater, R.A., Stead, L.F., Verghese, E.T., and Speirs, V. (2015). Down-Regulation of miR-92 in Breast Epithelial Cells and in Normal but Not Tumour Fibroblasts Contributes to Breast Carcinogenesis. PLoS ONE *10*, e0139698.

30. Bartel, D.P. (2009). MicroRNAs: target recognition and regulatory functions. Cell *136*, 215–233.

31. Yan, Q., Shen, C., Qin, J., Li, W., Hu, M., Lu, H., Qin, D., Zhu, J., Gao, S.J., and Lu, C. (2016). HIV-1 Vpr Inhibits Kaposi's Sarcoma-Associated Herpesvirus Lytic Replication by Inducing MicroRNA miR-942-5p and Activating NF-κB Signaling. J. Virol. *90*, 8739–8753.

32. Zhang, G., Liu, Z., Xu, H., and Yang, Q. (2016). miR-409-3p suppresses breast cancer cell growth and invasion by targeting Akt1. Biochem. Biophys. Res. Commun. *469*, 189–195.

33. Altomare, D.A., and Testa, J.R. (2005). Perturbations of the AKT signaling pathway in human cancer. Oncogene *24*, 7455–7464.

34. Hamidi, A., Song, J., Thakur, N., Itoh, S., Marcusson, A., Bergh, A., et al. (2017). TGF-β promotes PI3K-AKT signaling and prostate cancer cell migration through the TRAF6-mediated ubiquitylation of p85α. Sci. Signal *10*, eaal4186.

35. Qu, X., Shen, L., Zheng, Y., Cui, Y., Feng, Z., Liu, F., and Liu, J. (2014). A signal transduction pathway from TGF-β1 to SKP2 via Akt1 and c-Myc and its correlation with progression in human melanoma. J. Invest. Dermatol. *134*, 159–167.

36. Dong, H., Cao, W., and Xue, J. (2019). Long noncoding FOXD2-AS1 is activated by CREB1 and promotes cell proliferation and metastasis in glioma by sponging miR-185 through targeting AKT1. Biochem. Biophys. Res. Commun. *508*, 1074–1081.

37. Wu, H., Liu, H.Y., Liu, W.J., Shi, Y.L., and Bao, D. (2018). miR-377-5p inhibits lung cancer cell proliferation, invasion, and cell cycle progression by targeting AKT1 signaling. J. Cell. Biochem. *120*, 8120–8128.

38. Thiery, J.P., Acloque, H., Huang, R.Y.J., and Nieto, M.A. (2009). Epithelial-mesenchymal transitions in development and disease. Cell *139*, 871–890.

39. Chen, R., Liu, Y., Zhuang, H., Yang, B., Hei, K., Xiao, M., Hou, C., Gao, H., Zhang, X., Jia, C., et al. (2017). Quantitative proteomics reveals that long non-coding RNA MALAT1 interacts with DBC1 to regulate p53 acetylation. Nucleic Acids Res. *45*, 9947–9959.

OMTN, Volume *20*

Supplemental Information

circRNA-AKT1 Sequesters miR-942-5p to Upregulate AKT1 and Promote Cervical Cancer Progression

Rongying Ou, Laiming Mo, Huijing Tang, Shaolong Leng, Haiyan Zhu, Liang Zhao, Yi Ren, and Yunsheng Xu

Supplementary figure 1 Heat-map exhibited the expression of circRNAs in CC tissues and the matched adjacent non-cancerous tissues. (A-B) The heat-map displayed the expression of related circRNAs in CC tissues (n = 3) and the matched adjacent non-cancerous tissues (n = 3).

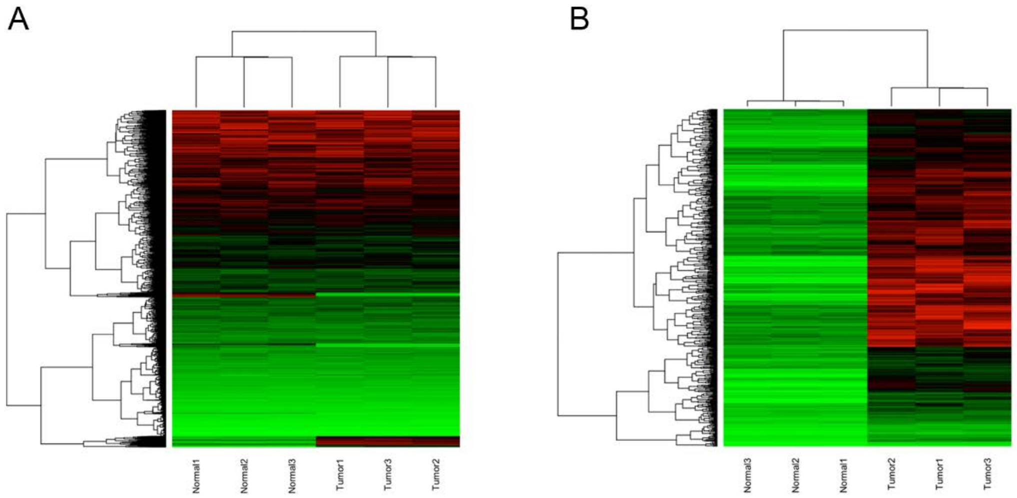

Supplementary figure 2 Northern blot measured the expression of AKT1 or circ-AKT1 in differently treated groups. (A) Northern blot assay detected the RNA expression of AKT1 and circ-AKT1 in RNase R treatment. (B) Northern blot assay detected the RNA expression of AKT1 with or without flanking intron treatment (Alu). GAPDH was negative control.

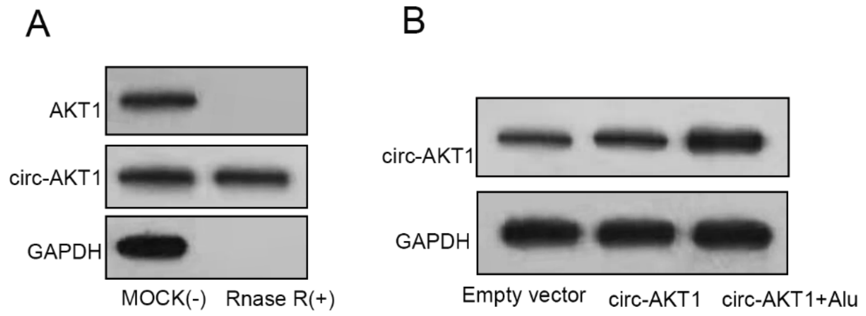

Supplementary figure 3 The expression of TGF-β in CC tissues and cells was examined. (A-B) RT-qPCR detected that the expression of TGF-β in CC tissues (n = 58) and cells as well as in corresponding control groups (n = 58). $^{**}P < 0.01$

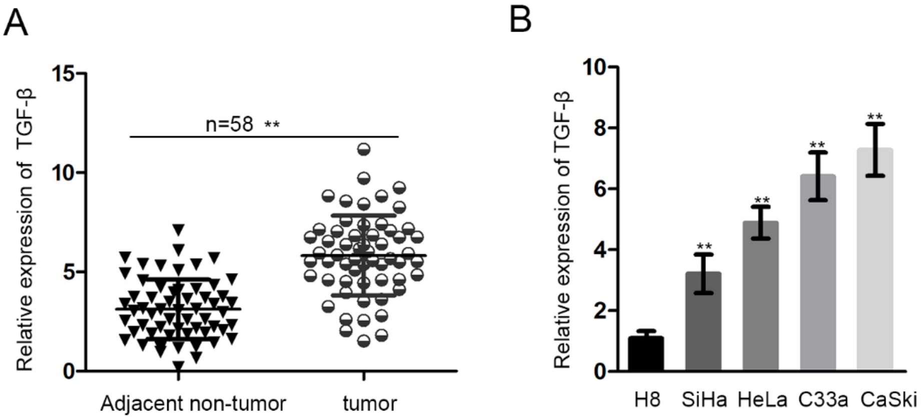

Table legends

Supplementary Table 1 Related sequences of genes were provided. Related PCR primer sequences and interference/overexpression sequences were exhibited.

Gene name:	PCR primer sequences:	Covergent primers for circ-AKT:	
circ-AKT1	F：CGTTCTTCTCCGAGTGCAG<br>R：ACGTTCTTCTCCGAGTGCAG	F：TGTTGAGGGTTGTCTCCGTG<br>R：GGACAGTCATGAGCTTCGCT	
miR-942-5p	F：CUUCUCUGUUUUGGCCAUGUG<br>R：CTCTACAGCTATATTGCCAGCCAC		
AKT1	F: CCTTCTTGAGCAGCCCTGAA<br>R: TACGAGATGATGTGCGGTCG		
**Plamids name:**	**Sequences:**		
siRNA	CUGACCAAGGUGGAAGAACAG		
circ-AKT1-siRNA#1	GUGGAAGAACAGCUUGGUCAG		
circ-AKT1-siRNA#2	CACCACCUGACCAAGCUGUUC		
circ-AKT1-siRNA#3	CACCUGACCAAGCUGUUCUUC		
si-AKT1#1	GCACCUUCAUUGGCUACAA		
si-AKT1#2	GGCCCAACACCUUCAUCAU		
si-AKT1#3	GGAGACUGACACCAGGUAU		
NC inhibitor	CACAUGGCCAAAACCUUCUCUA		
miR-942-5p inhibitor	CACAUGGCCAAAACAGAGAAGA		
NC mimics	UAGAGAAGGUUUUGGCCAUGUG		
miR-942-5p mimics	UCUUCUCUGUUUUGGCCAUGUG		

Supplementary Table 2 The accession number for the first 50 upregulated circRNAs in CC tissues was provided.

**circRNA**	**Accession number**				
circ-AKT1	NM_005163				
circ-DCLK2	NR_036614				
circ-CAMK2D	NM_001221				
circ-PLOD2	NM_000935				
circ-AGGF1	NM_018046				
circ-BBS9	NM_014451				
circ-EPB41	NM_004437				
circ-ANKS1B	NM_152788				
circ-TRIAP1	NM_016399				
circ-ANKRD36	NM_001164315				
circ-MBOAT2	NM_138799				
circ-KDM4B	NM_015015				
circ-HSF4	NM_001040667				
circ-EXOC6	NM_019053				
circ-PITPNB	NM_012399				
circ-PTPN12	NM_002835				
circ-PARD3	NM_019619				
circ-FGD5	NM_152536				
circ-GLB1	NM_000404				
circ-PTDSS1	NM_014754				
circ-PHACTR4	NM_023923				
circ-RAPGEF1	NM_005312				
circ-ZNF717	NM_001128223				
circ-PAN3	NM_175854				
circ-SLC4A5	NM_021196				
circ-RNPS1	NM_006711				
circ-HDAC11	NM_024827				
circ-PAPPA	NM_002581				
circ-EPB41L5	NM_020909				
circ-VCAN	NM_004385				
circ-PHKB	NM_000293				
circ-WSB1	NM_015626				
circ-TRIM37	NM_015294				
circ-DPY19L1	NM_015283				
circ-MAST2	NM_015112				
circ-AK127472	uc003qaq.1				
circ-NFAT5	NM_006599				
circ-SLMAP	NM_007159				
circ-ADAMTS6	NM_197941				
circ-MLLT6	NM_005937				
circ-NR3C2	NM_000901				
circ-KMT2E	NM_018682				
circ-SLC3A2	NM_002394				
circ-DOCK2	NM_004946				
circ-STXBP4	NM_178509				
circ-CANX	NM_001746				
circ-TRIO	NM_007118				
circ-FADS2	NM_004265				
circ-STARD7	NM_020151				
circ-NAV3	NM_014903				

1. Over-expressed loop-assisted sequence of circ-AKT1：

pcDNA3.1/ circ-AKT1

AGTAGAGACGGGGTTTCACCATGTTGGCCAGGCTGGTCTTCACTTTTTGTAAAGGTACGTACTAATGACTTTTTTTTTATACT
TCAGCTGTTCTTCCACCTGTCCCGGGAGCGTGTGTTCTCCGAGGACCGGGCCCGCTTCTATGGCGCTGAGATTGTGTCAGCCC
TGGACTACCTGCACTCGGAGAAGAACGTGGTGTACCGGGACCTCAAGGTGCGCTGGCGGGCAGGCAGGGGGGCAGGGCCCT
GGGGGCCTGGCGGCACTGACCTGAGGCCACCTTTCCCCTAGCTGGAGAACCTCATGCTGGACAAGGACGGGCACATTAAG
ATCACAGACTTCGGGCTGTGCAAGGAGGGGATCAAGGACGGTGCCACCATGAAGACCTTTTGCGGCACACCTGAGTACCT
GGCCCCCGAGGTGTGCGCCCCACCTGCGTGCATACGCGTTGCTGCGTCCCCACGTCCTGAGCACACGCAATGCTGTGTCC
TCTCTGTGCCCCAAGCACGTCACACCTCCCCCGGCAGTGTCCCGGACACCCCTTGATGCCGAGTCCTGCCCATCTGCCAC
CCGTGCAGGTGCTGGAGGACAATGACTACGGCCGTGCAGTGGACTGGTGGGGGCTGGGCGTGGTCATGTACGAGATGATG
TGCGGTCGCCTGCCCTTCTACAACCAGGACCATGAGAAGCTTTTTGAGCTCATCCTCATGGAGGAGATCCGCTTCCCGCG
CACGCTTGGTCCCGAGGCCAAGTCCTTGCTTTCAGGGCTGCTCAAGAAGGACCCCAAGCAGAGGTGAGGGCCGCCCATCC
CAGCTACAGGCTACACCTCCATCCCCTCATCCCCAGGCTGCACCTGCCCCTGCGCCAGGCGGTCCTGGCACCTCCCAGAC
TACACTGATAGCCAAAGCTTGATGTCCTTGGCCAGGGCTGATCTCCAAAGCCTCAGGCCCAAGCAGAGTGGCGGGCAGGC
CGGGGTCAGTTGGGCCTCTGTCCCCAACCCTGCAGCCTGCACCCACCCACTCAGGAAGCCCCTGCCCTGCCGTGAGCTCT
GTGGTGCTTTGCTACCCACAGCTGCTCAGGGACGCTGCACCACCGGCTCCCCTCCCTGGCCCCGGAACGTCCTGTCTGGC
GGGCCCTACATCACAGGAGGAAGGGGCCTGAACCCAGGGCCTGGGCAGGTGGCGGTACCGACACTGTGGCCTTGTTTCCT
GCCTGCAGGCTTGGCGGGGGCTCCGAGGACGCCAAGGAGATCATGCAGCATCGCTTCTTTGCCGGTATCGTGTGGCAGCA
CGTGTACGAGAAGAAGGTGCGGCTGCTCCCCGCATATTCACGCGCACGCATGCTCCCCACATATCCACACTCACGCATGC
ACGTGGCACGCTCGCCAGATTTCCCACACACTCGCCCTCACCTCAGGAGCCTGCTGCAGTCCTGGTACAAGGAGGGCCTT
GCTGCACCAACCTCAGCGCCTGGTGCTCAGAGGCTCTGGCACTGCCGGGTTCCACCAGGAAACTGGCCTGGTCCTCATTT
CCTCCTCCCCTCGGAGGTGTGTCACACTCTGAGTTTCCTCCTCCCCTCGGAGGTGTGTCACACTCTGAGTGCCAGCCTTG
GGGTTCCCTTCCCTGACGCTGTGCAGTGAAGGCTGGCTGGTGGTGGACCAGGGGTGCTGCCCCTTGGCCTCCACGAGTTC
CTCCTGTTTGACCTCAATCCCTTCTGCCGCAAGGAGAGCCCAGCCCTATTTCTGGCTGTGCAGGGACAGGGACAGCACCT
ACTTTTCTGGCACATGGGGGAGTCCGCCCTGGAGGGAGGCAGCTCTTTCTGCATGAGTCGCCATCCTGGGTGCTCATCTT
TCAGGGACCCTAGGAGCCCTGGCCATCACCCCAGCTGATGGGGTCTGCCAGCTGGGTTCGGAAGCCTGCACTCTGAGATG
CTGGGTGCCCTGTCACCAGGTGTGCTTCTGCCCCGTTCCCAGCCTGCCCCTCCCCTGAATGCCTGTCCTAGGCTGTGTGC
AGCTGGTCGGGCTGGGCAGCCCTGGGACCCAGCTGGGCACCTGTTCTCCCATTAACACAGGGTGCCCTGGTCCTGGTCCC
CGTTCCTGCCTTGAGGCCTGCCACCCTCCTGCCCTGCCCTCCAAGGGTATGGGGGGCCTGGGCAGTGCTCAACCTCTGCC
CAGCCTTGACCAGAGACCTTGCTAATTGACAATGGACAATGCTGGTGCCCAGCCCAGACTCTGGGGTGGGGCAGTGCTTG
AGGGGGTCCTTGACTGTGGGACCTGAGGGGCTCTCTGGCAGCTCCTCACGTGTGCACATCACCTTATAGTCACCCTTCAT
CCTGGGTCATTGAGAGTCCTGTCTGCAGCCAGATGCCAGCAGGCTGTGGTCCTGTAGTCCCTGAGCCATGGAGGCAGGGT
GGGCTCATCCTGTGCACCTTGTTCCACAGTCTCCCTCTTTCCCAATTTACAAGAAGACCAATAAGAAAAATAAGAAAACA
AGAAAGAAAAAGGAAAAATAAAGTTATCACCTCCTGGTAGCAGGGAGGGTCTCTCAGAGCTGGGCTGCACTACCTCCTGT
CGCCTGCTGGGGCTGCTGTGAGGGCAGCTTTGCGTCTCAGCTGTTGAGGGTTGTCTCCGTGCTCCTGCACCCCTCATGTC
CCTCCCTCCCAGAGCTGCCTCTGTGGGGGGCTGGTGGCTTGCTGCTCTCTGACATCAGTCCTGCCTGGAGACCCCTTGGA
GATCCAGGTGCTTTGAAGGTCTTGAGCACACTTGAGGGTGTGCTGGGAGTGGGGAGCGAAGCTCATGACTGTCCCGTCTG
CCCACCTCTGCAGCTCAGCCCACCCTTCAAGCCCCAGGTCACGTCGGAGACTGACACCAGGTATTTTGATGAGGAGTTCA
CGGCCCAGATGATCACCATCACACCACCTGACCAAGGTAAGAAGCAAGGAAAAGAATTAGAGACCAGCCTGGCCAACATGGTG
AAACCTTGTCTCTACT

2. Hsa_circ_0033550 circRNA Genomic Sequence:

CTGTTCTTCCACCTGTCCCGGGAGCGTGTGTTCTCCGAGGACCGGGCCCGCTTCTATGGCGCTGAGATTGTGTCAGCCCTGGACTACCTGCACTCGGAGAAGAACGTGGTGTACCGGGACCTCAAGGTGCGCTGGCGGGCAGGCAGGGGGGCAGGGCCCTGGGGGCCTGGCGGCACTGACCTGAGGCCACCTTTCCCCTAGCTGGAGAACCTCATGCTGGACAAGGACGGGCACATTAAGATCACAGACTTCGGGCTGTGCAAGGAGGGGATCAAGGACGGTGCCACCATGAAGACCTTTTGCGGCACACCTGAGTACCTGGCCCCCGAGGTGTGCGCCCCACCTGCGTGCATACGCGTTGCTGCGTCCCCACGTCCTGAGCACACGCAATGCTGTGTCCTCTCTGTGCCCCAAGCACGTCACACCTCCCCCGGCAGTGTCCCGGACACCCCTTGATGCCGAGTCCTGCCCATCTGCCACCCGTGCAGGTGCTGGAGGACAATGACTACGGCCGTGCAGTGGACTGGTGGGGGCTGGGCGTGGTCATGTACGAGATGATGTGCGGTCGCCTGCCCTTCTACAACCAGGACCATGAGAAGCTTTTTGAGCTCATCCTCATGGAGGAGATCCGCTTCCCGCGCACGCTTGGTCCCGAGGCCAAGTCCTTGCTTTCAGGGCTGCTCAAGAAGGACCCCAAGCAGAGGTGAGGGCCGCCCATCCCAGCTACAGGCTACACCTCCATCCCCTCATCCCCAGGCTGCACCTGCCCCTGCGCCAGGCGGTCCTGGCACCTCCCAGACTACACTGATAGCCAAAGCTTGATGTCCTTGGCCAGGGCTGATCTCCAAAGCCTCAGGCCCAAGCAGAGTGGCGGGCAGGCCGGGGTCAGTTGGGCCTCTGTCCCCAACCCTGCAGCCTGCACCCACCCACTCAGGAAGCCCCTGCCCTGCCGTGAGCTCTGTGGTGCTTTGCTACCCACAGCTGCTCAGGGACGCTGCACCACCGGCTCCCCTCCCTGGCCCCGGAACGTCCTGTCTGGCGGGCCCTACATCACAGGAGGAAGGGGCCTGAACCCAGGGCCTGGGCAGGTGGCGGTACCGACACTGTGGCCTTGTTTCCTGCCTGCAGGCTTGGCGGGGGCTCCGAGGACGCCAAGGAGATCATGCAGCATCGCTTCTTTGCCGGTATCGTGTGGCAGCACGTGTACGAGAAGAAGGTGCGGCTGCTCCCCGCATATTCACGCGCACGCATGCTCCCCACATATCCACACTCACGCATGCACGTGGCACGCTCGCCAGATTTCCCACACACTCGCCCTCACCTCAGGAGCCTGCTGCAGTCCTGGTACAAGGAGGGCCTTGCTGCACCAACCTCAGCGCCTGGTGCTCAGAGGCTCTGGCACTGCCGGGTTCCACCAGGAAACTGGCCTGGTCCTCATTTCCTCCTCCCCTCGGAGGTGTGTCACACTCTGAGTTTCCTCCTCCCCTCGGAGGTGTGTCACACTCTGAGTGCCAGCCTTGGGGTTCCCTTCCCTGACGCTGTGCAGTGAAGGCTGGCTGGTGGTGGACCAGGGGTGCTGCCCCTTGGCCTCCACGAGTTCCTCCTGTTTGACCTCAATCCCTTCTGCCGCAAGGAGAGCCCAGCCCTATTTCTGGCTGTGCAGGGACAGGGACAGCACCTACTTTTCTGGCACATGGGGGAGTCCGCCCTGGAGGGAGGCAGCTCTTTCTGCATGAGTCGCCATCCTGGGTGCTCATCTTTCAGGGACCCTAGGAGCCCTGGCCATCACCCCAGCTGATGGGGTCTGCCAGCTGGGTTCGGAAGCCTGCACTCTGAGATGCTGGGTGCCCTGTCACCAGGTGTGCTTCTGCCCCGTTCCCAGCCTGCCCCTCCCCTGAATGCCTGTCCTAGGCTGTGTGCAGCTGGTCGGGCTGGGCAGCCCTGGGACCCAGCTGGGCACCTGTTCTCCCATTAACACAGGGTGCCCTGGTCCTGGTCCCCGTTCCTGCCTTGAGGCCTGCCACCCTCCTGCCCTGCCCTCCAAGGGTATGGGGGGCCTGGGCAGTGCTCAACCTCTGCCCAGCCTTGACCAGAGACCTTGCTAATTGACAATGGACAATGCTGGTGCCCAGCCCAGACTCTGGGGTGGGGCAGTGCTTGAGGGGGTCCTTGACTGTGGGACCTGAGGGGCTCTCTGGCAGCTCCTCACGTGTGCACATCACCTTATAGTCACCCTTCATCCTGGGTCATTGAGAGTCCTGTCTGCAGCCAGATGCCAGCAGGCTGTGGTCCTGTAGTCCCTGAGCCATGGAGGCAGGGTGGGCTCATCCTGTGCACCTTGTTCCACAGTCTCCCTCTTTCCCAATTTACAAGAAGACCAATAAGAAAAATAAGAAAACAAGAAAGAAAAAGGAAAAATAAAGTTATCACCTCCTGGTAGCAGGGAGGGTCTCTCAGAGCTGGGCTGCACTACCTCCTGTCGCCTGCTGGGGCTGCTGTGAGGGCAGCTTTGCGTCTCAGCTGTTGAGGGTTGTCTCCGTGCTCCTGCACCCCTCATGTCCCTCCCTCCCAGAGCTGCCTCTGTGGGGGGCTGGTGGCTTGCTGCTCTCTGACATCAGTCCTGCCTGGAGACCCCTTGGAGATCCAGGTGCTTTGAAGGTCTTGAGCACACTTGAGGGTGTGCTGGGAGTGGGGAGCGAAGCTCATGACTGTCCCGTCTGCCCACCTCTGCAGCTCAGCCCACCCTTCAAGCCCCAGGTCACGTCGGAGACTGACACCAGGTATTTTGATGAGGAGTTCACGGCCCAGATGATCACCATCACACCACCTGACCAAG
